# Functional Properties of POU1F1 Mutants in the Transcriptional Regulation of the Thyrotropin β Gene Compared with the Prolactin Gene

**DOI:** 10.3390/ijms27010119

**Published:** 2025-12-22

**Authors:** Yuto Kawauchi, Shigekazu Sasaki, Akio Matsushita, Hiroko Misawa Nakamura, Miho Yamashita, Keisuke Kakizawa, Kenji Ohba, Daisuke Tsuriya, Tomohiro Tanaka, Takafumi Suda

**Affiliations:** 1 Second Division, Department of Internal Medicine, Hamamatsu University School of Medicine, 1-20-1 Handayama, Chuo-ku, Hamamatsu 431-3192, Shizuoka, Japan; 41240030@hama-med.ac.jp (Y.K.);; 2 Department of Internal Medicine, Nagoya City University Mirai Kousei Hospital, 1501-2 Segobo, Meito-ku, Nagoya 465-8650, Aichi, Japan; 3 Internationalization Center, Hamamatsu University School of Medicine, 1-20-1 Handayama, Chuo-ku, Hamamatsu 431-3192, Shizuoka, Japan; 4 Medical Education Center, Hamamatsu University School of Medicine, 1-20-1 Handayama, Chuo-ku, Hamamatsu 431-3192, Shizuoka, Japan; 5 Department of Gastroenterology and Metabolism, Nagoya City University Graduate School of Medical Sciences, 1 Kawasumi, Mizuho-cho, Mizuho-ku, Nagoya 457-8601, Aichi, Japan

**Keywords:** POU1F1, GATA2, transcription, protein stability, combined pituitary hormone deficiency, thyroid-stimulating hormone (TSH), prolactin, growth hormone (GH), recombinant GH, hypothalamus–pituitary–thyroid axis

## Abstract

Mutations in the POU1F1 gene cause defects in the expression of the genes encoding thyroid-stimulating hormone (TSH)-β subunit, growth hormone (GH), and prolactin (PRL). Here, we characterized 15 missense and nonsense mutations. Protein stability was reduced in the P14L, P24L, F135C, K145X, F233S and E250X mutants. Transactivation by 15 mutants in the TSHβ promoter was moderately correlated with that of the PRL promoter. Based on their transcriptional activity, we classified them into three groups: group I, equivalent to the wild type; group II, partial; and group III, substantially lost. A review of case reports on four patients with group II mutations revealed that TSH deficiency manifested after recombinant GH therapy. A transcription factor, GATA2, is the main activator in the TSHβ gene, while POU1F1 protects its function from inhibition by the suppressor region (SR). We found that the SR is critical for the pathogenesis of TSH deficiency. The transactivation of the TSHβ promoter by the K216E mutant was equivalent to that of wild-type POU1F1; however, that of the PRL promoter was low, while the opposite was found in the R271W mutant. The functional property of K216E suggests that the interaction of POU1F1 with GATA2 may not always be necessary for the activation of the TSHβ promoter.

## 1. Introduction

**Figure 1:**
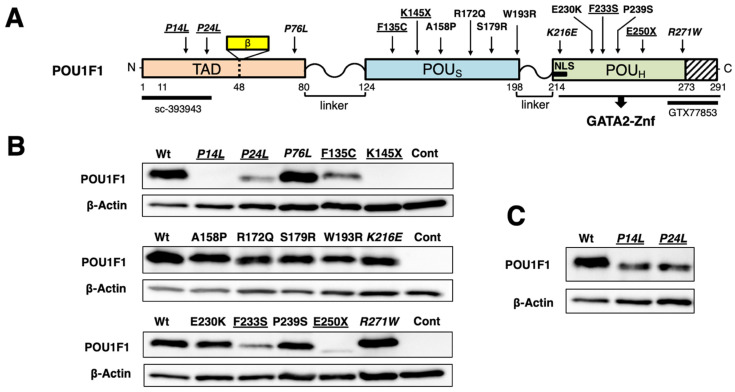
(**A**) Schematic representation of wild-type human POU1F1 protein and its 15 mutants. N-terminal transactivation domain (TAD), POU-specific domain (POUs), and POU homeodomain (POU_H_) are indicated. POU_H_ interacts with the zinc finger domain of GATA2 (GATA2-Znf). The positions of the POU1F1β variant and nuclear localization signal (NLS) are indicated. (**B**,**C**) Protein levels of mutant POU1F1s. Western blots were performed with a whole-cell extract of CV-1 cells transfected with equal amounts (μg) of expression plasmids for various mutants using antibodies against the N-terminus (sc-393943) (**B**) and the C-terminus (GTX77853) (**C**). The POU1F1 mutants for which protein levels were reduced are underlined. The mutants for which inheritance is reported to be heterozygotic are indicated in italic.

**Figure 2:**
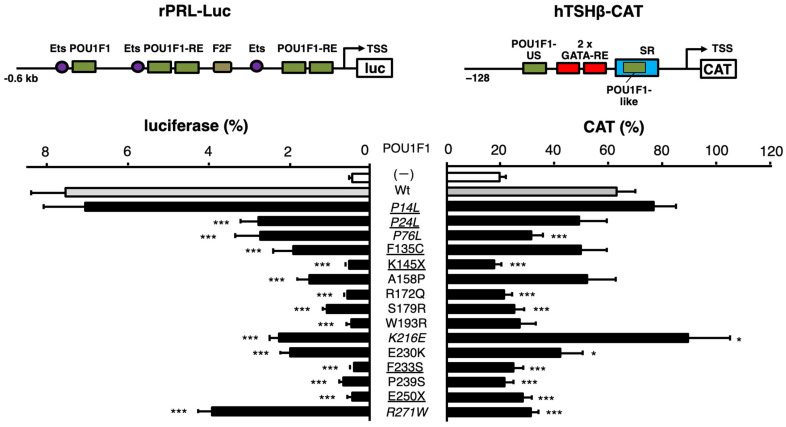
POU1F1 mutations cause various degrees of defects in the transcriptional activity of the rat PRL and human TSHβ promoters. Schematic representation of rPRL-Luc (**top left**) and hTSHβ-CAT (**top right**). Ets, binding site for E26 transformation-specific transcription factor (Ets); POU1F1-RE, POU1F1-responsive element; TSS, transcription start site; GATA-RE, GATA-responsive element; POU1F1-US and POU1F1-like, POU1F1-REs upstream and downstream of 2 GATA-REs, respectively; SR, suppressor region. rPRL-Luc was transfected into HeLa cells along with pCMV-β-galactosidase (pCMV-GAL) and expression plasmids for wild-type POU1F1 (pCB6^+^-hPIT1) or its mutants (**bottom left**). Likewise, hTSHβ-CAT was transfected into CV-1 cells along with pCMV-GAL and expression plasmids for GATA2 (pcDNA3-mGATA2) and wild-type or mutant POU1F1 (**bottom right**). The cells were exposed to Lipofectamine 3000 reagent for 24 h. After additional incubation for 24 h, the cells were harvested. Luciferase and CAT activity was normalized with β-galactosidase activity. For each reporter assay, we performed transfection with pRSV-Luc or pCMV-CAT, the magnitudes of which were adjusted to a value of 100%. Each luciferase and CAT assay was performed in duplicate more than three times. Statistical significance was examined using analysis of variance and Fisher’s protected least significant difference test. * *p* < 0.05, *** *p* < 0.0005, compared with wild-type POU1F1. The POU1F1 mutants whose protein levels were reduced (Figure 1B,C) are underlined. The mutants for which inheritance was reported to be heterozygotic are indicated in italic.

POU domain transcription factor POU1F1 (previously known as Pit-1 or GHF-1) is essential for the differentiation of thyrotrophs, somatotrophs, and lactotrophs in the anterior pituitary gland [1] and transcriptionally activates (transactivates) the genes encoding thyroid-stimulating hormone-β subunit (TSHβ), growth hormone (GH), and prolactin (PRL) via their POU1F1-responsive elements (POU1F1-REs). POU1F1 also maintains the expression of other genes [2,3,4,5]. The POU1F1 protein consists of a transactivation domain (TAD), POU-specific domain (POU_S_), and POU homeodomain (POU_H_) (Figure 1A). The POU_S_ and POU_H_ domains recognize the ATTC and AT sequences, respectively, in the canonical POU1F1-RE [6,7,8]. The POU1F1 protein is known to have structural flexibility [9] and interacts with various transcription factors in its target genes [4,10,11]. In the rat PRL promoter, POU1F1 exists not only as a monomer but also as a dimer on its POU1F1-RE [12] and interacts with estrogen receptor α [13]. In the rat (Figure 2, top left) and human PRL genes, it is also associated with E26 transformation-specific transcription factor (Ets) [13,14]. The cooperation of POU1F1 with Ets is determined by the number of nucleotides spaced between the POU1F1-RE and the Ets-binding site [15]. POU1F1 may modify the chromatin structure and the transcription rate of this gene [13] by recruiting multiple cofactors [16,17]. Furthermore, its phosphorylation [18] has various effects on its function [19,20]. 

According to Jadhav et al. [21], 25 missense, seven nonsense, and seven frameshift mutations have been identified in the human POU1F1 gene. Clinically, these mutations cause POU1F1-related combined pituitary hormone deficiency (CPHD), including defects in the production of GH (GHD), TSH (TSHD), and PRL (PRLD) [22]. The identification of this triad provides important guidance for the discovery of POU1F1 abnormalities. However, these combinations are not always observed simultaneously. The symptoms of TSHD, including prolonged jaundice, feeding difficulties, and failure to thrive, may occur in the neonatal period. In contrast, short stature caused by GHD is usually noticed at least several months to two years after birth. PRLD is often detected in the screening of pituitary hormones in patients in whom CPHD is clinically suspected, while PRLD itself may be manifested as insufficient lactation in adult women after delivery [23,24]. Thus, the first symptom is thought to be TSHD in typical cases with a POU1F1 mutant, where its function is completely lost. However, in some cases with naturally occurring POU1F1 mutations, TSHD is detected after the diagnosis of GHD—that is, several months or years after birth [21,25,26,27,28,29,30]. One reason for this may be that the diagnosis of central hypothyroidism, including TSHD caused by POU1F1 mutations, is not always easy [31,32]. Another possibility is that recombinant GH (rGH) administration may increase triiodothyronine (T3) levels in thyrotrophs, because GH has been reported to activate the type 2 deiodinase (D2) gene [33], resulting in a decrease in TSH secretion. Therefore, the symptom of mild TSHD [34,35,36,37], which is partially compensated for by a negative feedback mechanism via the hypothalamus–pituitary–thyroid (H-P-T) axis, may be manifested after rGH treatment.

To exclude the influence of T3, thyroxine (T4), and thyrotropin-releasing hormone (TRH), an experimental system using cultured cells is necessary. However, investigations of TSHβ expression using cultured cells are limited. TtT97, a TSH-producing tumor, requires 4–9 months to grow [38]. TαT1 cells were established from transgenic mice in which the SV40 large T antigen gene was driven by the chorionic gonadotropin α (CGA) subunit promoter [39]. However, their transfection efficiency was quite low [40]. The function of four POU1F1 mutants was examined in the context of the TSHβ promoter using non-thyrotroph cells, including JEG3 [41], αT3 [42], 293 [43], and 293T cells [32]. However, the functions of the various mutants were not compared under common experimental conditions; these studies characterized individual POU1F1 mutants, using the wild type as a positive control.

The coexistence of another transcription factor, GATA2, with POU1F1 is essential not only for TSHβ expression [10,44] but also for thyrotroph differentiation in the pituitary gland [45]. In vitro experiments suggest that POU1F1 associates with GATA2 via protein–protein interactions [44,46] (Figure 1A). Two GATA response elements (GATA-REs) are present in the human TSHβ promoter, with a POU1F1-RE located at the 5’ end and another at the 3’ end of these GATA-REs (Figure 2, top right) [10]. We designated the one at the 5’ end (upstream) POU1F1-US (previously Pit1-US) and the one at the 3’ end the POU1F1-like (previously Pit1-like) sequence [6]. Due to the lack of suitable cell lines, we employed kidney-derived CV-1 cells, which have often been used in studies on T3-dependent positive and negative regulation [47]. This reconstitution system suggested that the number of spacing nucleotides between POU1F1-US and GATA-REs influences the cooperation between POU1F1 and GATA2 [6], as in the case with POU1F1 and Ets in the PRL promoter [48,49,50], and that the deletion of the sequence that encompasses the POU1F1-like sequence rendered GATA2 able to fully activate the TSHβ promoter without POU1F1. We designated this sequence the suppressor region (SR) and found that POU1F1 competes with the putative SR-binding protein (SRBP) (Figure 2, top right) [6]. Although the biochemical details of SRBP have not been determined yet, this finding suggests that the main transcriptional activator for the TSHβ gene is GATA2, while POU1F1 is necessary to protect GATA2 function from SRBP-induced suppression by competition on the SR (de-repression) [6].

In this study, we initially examined the protein stability of 15 POU1F1 mutants reported previously. Next, we hypothesized that the function of mutant POU1F1s in the TSHβ gene may be different from that of the PRL and GH genes because of its unique transcriptional regulation, especially via the SR. To examine the levels of transcriptional defects caused by these 15 mutants in the human TSHβ promoter, we compared them with those of a rat PRL promoter construct for three reasons. First, because the GH gene harbors numerous POU1F1-REs in its locus-controlling region (LCR), analysis using a reporter assay was predicted to be complicated. Second, previous studies have reported that the transcriptional activity of several POU1F1 mutants, including P14L, P24L, F135C, A158P, K216E, E230K, E250X, and R271W, in the PRL promoter is generally correlated with that in the GH promoter construct, which contains a single POU1F1-RE [51,52,53,54,55]. Third, functional analyses of POU1F1 were gathered in the context of the rat PRL gene (Figure 2, top left) using HeLa cells, which express low but detectable endogenous Ets [48,49,50]. Because the properties of R271W and K216E are unique among group II mutants, we examined their DNA binding using gel shift assays with the POU1F1-REs in the rat PRL and human TSHβ genes, and we analyzed the protein–protein interaction of the K216E mutant and GATA2 through glutathione S-transferase (GST) pulldown assays. All analyses in this study were performed in vitro but not in silico.

## 2. Results

### 2.1. Among 15 POU1F1 Mutants, Six Had Reduced Protein Stability

Figure 1A shows the locations of the 15 POU1F1 missense and nonsense mutations examined in the present study. We transfected equal amounts (μg) of the expression plasmids of these mutants into CV-1 cells and examined the protein levels of each mutant by Western blotting using whole-cell extracts. As shown in Figure 1B, wild-type POU1F1 (calculated Mr. of 32 kDa) was detected using an antibody (sc-393943) raised against the N-terminus of POU1F1. The protein levels of P14L and P24L were below detection and moderately reduced, respectively. As these N-terminal mutations may affect recognition by the antibody raised against this amino acid sequence (Figure 1A), Western blotting was performed using an antibody against the C-terminus (GTX77853). The protein expression of both P14L and P24L was again detected (Figure 1C); however, both signals were lower than those of wild-type POU1F1. We also observed reduced expression of the F135C and F233S proteins. E250X was detected as a faint signal at a position corresponding to a calculated Mr. of 28 kDa. Another truncation mutant, K145X, was predicted to have a low molecular weight (calculated Mr. of 16 kDa); however, we were unable to detect the protein signal at the corresponding position (Supplementary Data). Hence, we considered that the protein level of K145X had also decreased. Collectively, the protein stability of six out of 15 mutants was thought to be reduced.

### 2.2. Transactivation of the TSHβ Promoter by 15 Mutants Was Moderately Correlated with That of the PRL Promoter

**Figure 3:**
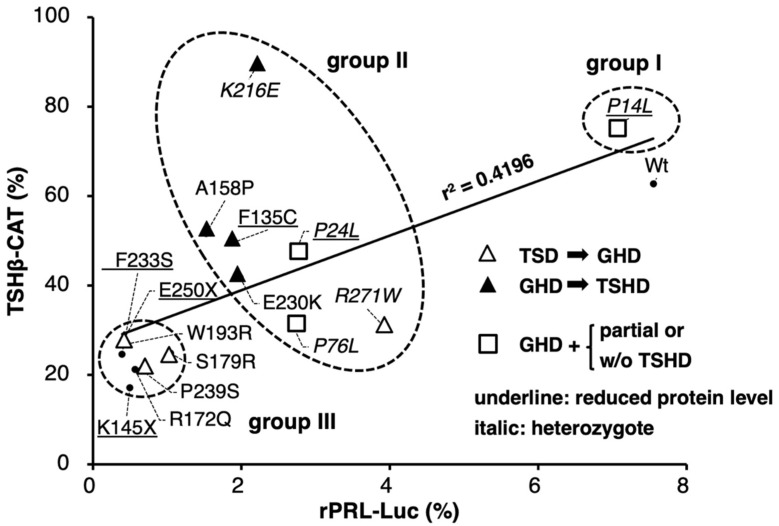
Scattergram of wild-type and 15 POU1F1 mutants showing the relationship of rPRL-Luc vs. hTSHβ-CAT activity based on the data in Figure 2. The solid line is the regression line. The POU1F1 mutants whose protein levels were reduced (Figure 1B,C) are underlined. The mutants whose inheritance is reported to be heterozygotic are indicated in italic. We classified P14L as group I, mutants with moderate transcriptional activity (P24L, P76L, F135C, A158P, K216E, E230K, R271W) as group II, and mutants with severely reduced activity (K145, R172Q, S179R, W193R, F233S, P239S, E250X) as group III. These three groups are circled with broken lines. GHD, GH deficiency; TSHD, TSH deficiency. Open triangles indicate the mutant POU1F1s, the cases in which TSHD was initially diagnosed and then GHD was detected. Closed triangles indicate mutant POU1F1s, the cases in which GHD was diagnosed first and then TSHD was detected. Open squares indicate the mutant POU1F1s, the cases in which GHD with partial TSHD (P24L) or isolated GHD (P76L) were reported.

Because firefly luciferase cDNA itself mediates artificial T3-dependent negative regulation [56], we have utilized a chloramphenicol acetyltransferase (CAT)-based reporter construct (hTSHβ-CAT) in our studies of TSHβ gene transcription [47]. Here, equal amounts (μg) of the expression plasmids for mutant POU1F1s were co-transfected with those for GATA2 and hTSHβ-CAT into CV-1 cells. We compared their activity with that of a luciferase gene fused to the rat PRL promoter (rPRL-Luc) co-transfected with POU1F1 mutants into HeLa cells [48,49,50]. The mutant POU1F1s showed various degrees of reduction in the transactivation of these two promoters (Figure 2). The scattergram in Figure 3 indicates that the transactivating function of these mutants was moderately correlated (r^2^ = 0.4196). Among the mutants with decreased protein levels (underlined in Figure 3), F233S and the truncated mutants, K145X and E250X, showed reduced activity for both promoters, whereas F135C and P24L exhibited moderate activity. Unexpectedly, P14L showed activity equivalent to that of the wild type in both promoters, despite a reduction in the protein level (Figure 1C).

### 2.3. Fifteen POU1F1 Mutants Were Classified into Three Groups Based on Their Transactivation Function

Based on the scattergram in Figure 3, we classified P14L as group I, those with moderate transcriptional activity (P24L, P76L, F135C, A158P, K216E, E230K, and R271W) as group II, and those with severely reduced activity (K145, R172Q, S179R, W193R, F233S, P239S, and E250X) as group III. With regard to group II and III mutants, we were interested in the time course of the clinical onset (or diagnosis) of TSHD, GHD, and PRLD. TSHD was first detected in case reports of group III mutants, including S179R, F233S, and P239S (open triangles). However, in group II mutants, including F135C, A158P, K216E, and E230K (solid triangles), GHD was initially observed, whereas TSHD was detected several months or years later. In these patients, TSHD was diagnosed after treatment with rGH. There is a possibility that TSHD, partially compensated for via the H-P-T axis, was rendered overt by the administration of rGH, which is known to suppress TSH production in the pituitary gland [34,35,36,37]. Although patients with P76L [57] were reported to have isolated GHD (open squares), the free T4 levels of patients in this group were at the lower limit of the normal range, suggesting the existence of mild TSHD.

### 2.4. Transactivation of the TSHβ Gene by the K216E Mutant Was Equivalent to That of the Wild Type, but That of the PRL Gene Was as Low as That of the Mutants in Group III, While the Opposite Was Found in the R271W Mutant

The scattergram of rPRL-Luc vs. hTSHβ-CAT (Figure 3) highlights the unique properties of R271W [41] and K216E [58] among the group II mutants. The transactivation of the rat PRL promoter by K216E was as low as that by F135C, A158P, and E230K, while that of the TSHβ promoter by this mutant was more pronounced and even slightly higher than that by wild-type POU1F1 (Figure 2 and Figure 3). Clinically, a patient with K216E was initially diagnosed with GHD and partial PRLD, whereas TSHD was detected several years later [58]. Conversely, the transcriptional activity of hTSHβ-CAT by R271W was almost the lowest, but that of rPRL-Luc was the highest among the group II mutants (Figure 3). In support of this, the proband in the case with R271W was diagnosed with severe hypothyroidism as the initial symptom [59]. An R271W mutation was later found in numerous patients and was regarded as the “hot spot” mutation [60,61]. A previous report [54] indicated that the transactivation level of R271W in the PRL promoter was similar to that in the GH promoter in GH3 cells. We reviewed 11 case reports on R271W, where the onset periods of TSHD and GHD were described. In six cases, TSHD was first identified before GHD [41,60,62,63,64,65], and, in two cases, TSHD was diagnosed simultaneously with GHD, although their case reports [66,67] indicate the possibility that TSHD symptoms were overlooked. This percentage is in contrast to other group II mutations, including F135C, A158P, E230K, and K216E, in which TSHD is detected only after GHD diagnosis and/or rGH treatment.

### 2.5. SR Is More Critical for the Pathogenesis of TSHD by POU1F1 Mutants than POU1F1-US

**Figure 4:**
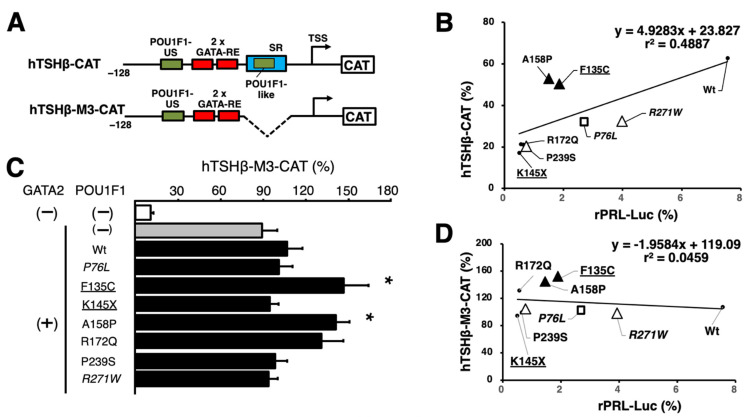
(**A**) Schematic representation of hTSHβ-CAT (**top**) and hTSHβ-M3-CAT (**bottom**). POU1F1-RE, POU1F1-responsive element; TSS, transcription start site; POU1F1-US, POU1F1-RE upstream; GATA-RE, GATA-responsive element; POU1F1-like, POU1F1-RE-like sequence downstream of 2 GATA-REs; SR, suppressor region. SR including POU1F1-like sequence was deleted in hTSHβ-M3-CAT. (**B**) Scattergram of wild type and seven selected POU1F1 mutants showing the relationship of hTSHβ-CAT vs. rPRL-Luc activity. The data for hTSHβ-CAT and rPRL-Luc are selected from those in Figure 2 and Figure 3. The activity between these two reporter genes is well correlated. Meanings of open triangles, closed triangles, and open squares are the same as in Figure 3. The POU1F1 mutants in which protein levels were reduced (Figure 1B) are underlined. The mutants for which inheritance is reported to be heterozygotic are indicated in italic. (**C**) hTSHβ-M3-CAT was transfected into CV-1 cells along with pCMV-GAL and expression plasmids for GATA2 (pcDNA3-mGATA2) and wild-type POU1F1 (pCB6^+^-hPIT1) or its seven selected mutants. CAT activity was normalized with β-galactosidase activity. For each reporter assay, we performed transfection with pCMV-CAT, the magnitude of which was adjusted to a value of 100%. Each CAT assay was performed in duplicate more than three times. Statistical significance was examined using analysis of variance and Fisher’s protected least significant difference test. *, *p* < 0.05, compared with wild-type POU1F1 (Wt). (**D**) Scattergram of wild type and seven selected POU1F1 mutants showing the relationship of hTSHβ-M3-CAT vs. rPRL-Luc activity. The data for rPRL-Luc are the same as in Figure 2 and Figure 3, and 4B. There is no correlation in activity between these two reporter genes. Meanings of points are the same as in Figure 3 and Figure 4B.

We re-examined the significance of the SR in the context of mutant POU1F1s. We selected seven mutants in groups II and III, in addition to the wild-type POU1F1. As in the case with Figure 3, the transactivation of hTSHβ-CAT was again moderately correlated with that of rPRL-Luc (r^2^ = 0.4887, Figure 4B). However, the deletion of the SR (hTSHβ-M3-CAT, Figure 4A) rendered GATA2 alone fully active, even in the absence of the wild-type or mutant POU1F1 (Figure 4C). Indeed, no correlation was observed between hTSHβ-M3-CAT and rPRL-Luc (r^2^ = 0.0459, Figure 4D). Thus, in the pathogenesis of TSHD in POU1F1-related CPHD, DNA binding with the POU1F1-like sequence in the SR plays a more critical role than that with POU1F1-US.

### 2.6. DNA Binding of Mutant POU1F1s with POU1F1-REs in the PRL and TSHβ Genes

**Figure 5:**
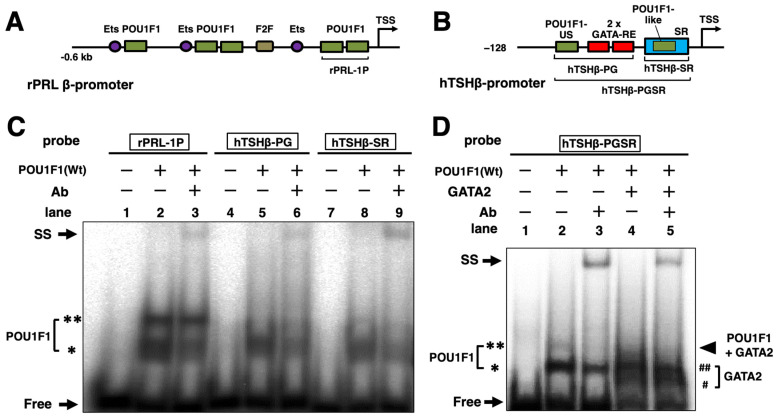
DNA binding of wild-type POU1F1 on the POU1F1-REs of the rat PRL (rPRL-1P) and human TSHβ promoter. (**A**,**B**) Schematic representation of the rat PRL (**A**) and human TSHβ promoter (**B**). Ets, binding site for E26 transformation-specific transcription factor (Ets). SR; suppressor region. POU1F1, POU1F1-RE; rPRL-1P, proximal 2 POU1F1-REs of the rat PRL gene. The positions of oligo-DNAs for the gel shift assays are indicated. hTSHβ-PG, containing POU1F1-US and two GATA-REs; hTSHβ-SR, containing the POU1F1-like sequence and SR; hTSHβ-PGSR, containing the sequence from POU1F1-US to the SR. (**C**) Gel shift assays demonstrated a wild-type human POU1F1 monomer (*) and dimer (**) on rPRL-1P (**left**) and POU1F1 monomers (*) on hTSHβ-PG (**middle**) and hTSHβ-SR (**right**). (**D**) Gel shift assays demonstrated a wild-type POU1F1 monomer (*), dimer (**), and complex of POU1F1 with GATA2 on hTSHβ-PGSR. #, GATA2 monomer; ##, GATA2 dimer. ^32^P-radiolabeled rPRL-1P, hTSHβ-PG, hTSHβ-SR, and hTSHβ-PGSR were incubated with a nuclear extract of CV-1 cells transfected with human POU1F1 and/or mouse GATA2 expression plasmids. These bands were specifically super-shifted (SS) when the POU1F1 protein was mixed with the anti-POU1F1 antibody (Ab, GTX77853) before incubation with ^32^P-radiolabeled probes. Free, free radiolabeled probes.

**Figure 6:**
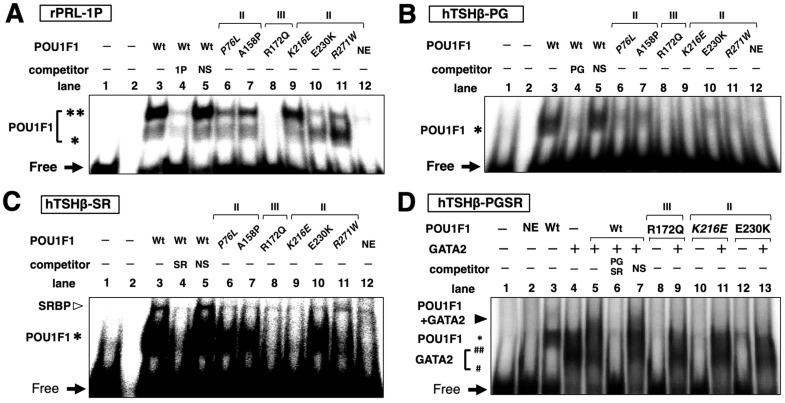
Gel shift analysis of mutant POU1F1s (P76L, A158P, R172Q, K216E, E230K, R271W) using ^32^P-radiolabeled rPRL-1P (**A**), hTSHβ-PG (**B**), hTSHβ-SR (**C**), and hTSHβ-PGSR (**D**). SR; suppressor region; POU1F1-US, POU1F1-RE upstream of 2 GATA-REs; POU1F1-like, POU1F1-RE-like sequence in SR; rPRL-1P, proximal 2 POU1F1-REs of the rat PRL gene; hTSHβ-PG, containing POU1F1-US and two GATA-REs; hTSHβ-SR, containing the POU1F1-like sequence and SR; hTSHβ-PGSR, containing the sequence from POU1F1-US to the SR. The positions of these probes are indicated in Figure 5. In each gel, the signal for wild-type POU1F1 was detected (lane 3), which was attenuated by cold oligonucleotides specific to the probe (lane 4 in (**A**–**C**) and lane 6 in (**D**)) but not by non-specific ones (NS, lane 5 in (**A**–**C**) and lane 7 in (**D**)). SRBP, suppressor region binding protein; NE, nuclear extract of non-transfected CV-1 cells; *, POU1F1 monomer; **, POU1F1 dimer; #, GATA2 monomer; ##, GATA2 dimer. Open arrowhead in (**C**), SRBP on SR; closed arrowhead in (**D**), complex of POU1F1 with GATA2 on hTSHβ-PGSR; Free, free radiolabeled probes. The mutants for which inheritance is reported to be heterozygous (P76L, K216E, and R271W) are indicated in italic.

Using gel shift assays, we attempted to examine the DNA binding of some of the mutant POU1F1s whose transcriptional activity was affected (Figure 3) without a reduction in protein levels (Figure 1B). As reported previously, wild-type POU1F1 binds POU1F1-RE (rPRL-1P) in the rat PRL promoter (Figure 5A) as a monomer and dimer [12], the bands of which were super-shifted with the anti-POU1F1 antibody (Figure 5C). In the human TSHβ gene (Figure 5B), POU1F1 binds POU1F1-US and POU1F1-like in the SR, whereas GATA2 binds two GATA-REs. We utilized probes designated as hTSHβ-PG, containing POU1F1-US and two GATA-REs; hTSHβ-SR, containing POU1F1-like and the SR; and hTSHβ-PGSR, containing the sequence from POU1F1-US to the SR. We detected specific bands that were super-shifted by the antibody (Figure 5C,D). In hTSHβ-PGSR, a faint band corresponding to the complex consisting of POU1F1 and GATA2 was observed when the GATA2 protein was added. We examined the DNA binding of a group I mutant, R172Q, and the group II mutants (P76L, A158P, K216E, E230K, and R271W). The results are shown in Figure 6. Specific bands that were competed for by cold oligonucleotides were detected. In rPRL-1P, monomer and dimer formation was reduced in the mutants, except for R271W and K216E (Figure 6A). As reported previously [54], the R271W mutant showed increased binding as a monomer, but not as a dimer. Consistent with the reduced activity of the TSHβ promoter by the R271W mutant (Figure 3), its DNA binding with hTSHβ-PG and hTSHβ-SR was reduced (Figure 6B,C). In spite of the potent transactivation of the hTSHβ gene by K216E, its DNA binding with hTSHβ-PG and hTSHβ-SR was also attenuated (Figure 6B,C), and complex formation with GATA2 on hTSHβ-PGSR was not observed (Figure 6D). As shown in Figure 6C, we also detected signals for SRBP (e.g., lane 3). Among the six mutants examined, the signal of SRBP was almost abolished in the presence of K216E (lane 9), while it was relatively prominent in the presence of R271W (lane 11).

### 2.7. Protein–Protein Interaction of K216E with GATA2 Was Reduced in Spite of Its Strong Transactivation Function in the hTSHβ Gene

**Figure 7:**
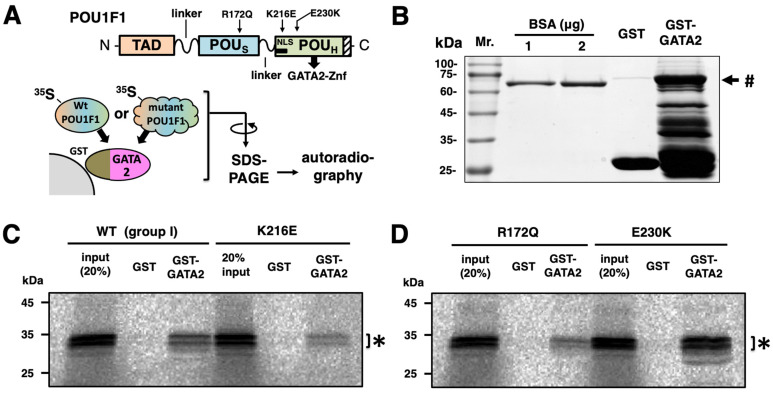
The protein–protein interaction of wild-type and mutant POU1F1s. (**A**) Schematic representation of human POU1F1 protein and its 3 mutants (**top**). N-terminal transactivation domain (TAD), POU-specific domain (POUs), and POU homeodomain (POU_H_) are indicated. K216E and E230K are located in the POU_H_ domain, which is known for protein–protein interactions with GATA2-Znf, while R172Q is located in the POU_S_ domain. The experimental procedure of the GST pulldown assay is indicated (**bottom**). GST-GATA2 fusion protein was incubated with ^35^S-methionine-labeled wild-type POU1F1 or mutants and analyzed using sodium dodecyl sulfate–polyacrylamide gel electrophoresis (SDS-PAGE) and autoradiography. (**B**) The preparation of the GST-GATA2 protein stained with Coomassie blue. #, GST-GATA2 fusion protein. The amount of GST-GATA2 protein was estimated with bovine serum albumin (BSA). (**C**) The protein–protein interaction of K216E was reduced. Specific signals (*) for wild-type POU1F1 or the K216E mutant were detected in the lanes for GST-GATA2 but not GST. The smaller-molecular-weight bands represent the translation products from the second methionine at codon 27. (**D**) Likewise, the interactions of the GST-GATA2 fusion protein with ^35^S-methionine-labeled R172Q or E230K mutants were analyzed. *, bands for R172Q or E230K mutant.

Previous deletion mapping [44,45] demonstrated that POU1F1 interacts with the zinc finger domain of GATA2 (GATA2-Znf) via the POU_H_ domain. Since codon 216 is located in the POU_H_ domain (Figure 1A and Figure 7A), we tested its protein–protein interaction with GATA2 using a GST pulldown assay. We also tested E230K, a mutant in the POU_H_ domain, and R172Q, the DNA binding of which was reduced in the context of rPRL-1P (Figure 6A) and hTSHβ (Figure 6B–D). The full-length GATA2 was fused to GST (Figure 7B) and incubated with ^35^S-methionine-labeled POU1F1 prepared using a rabbit reticulocyte system. The interaction of K216E and R172Q with GATA2 was reduced, whereas that of E230K was maintained (Figure 7C,D). Although we failed to clarify the mechanism by which the K216E mutant can activate the TSHβ promoter, the finding that this mutant was able to activate it in spite of a defect in physical association with GATA2 suggests that this interaction [44,45] may not always be essential for the transactivation of the TSHβ gene. Although the R172Q mutation was not located in the POU_H_ domain (Figure 7A), its interaction with GATA2 was impaired, probably due to the global conformational alteration by this mutation.

## 3. Discussion

**Figure 8:**
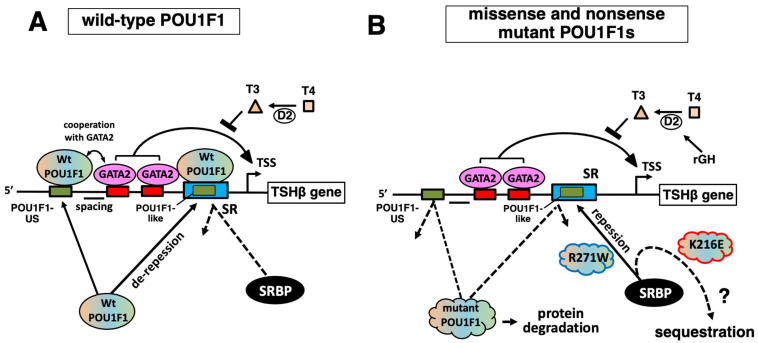
Multiple defects are involved in the pathophysiology of TSH deficiency (TSHD) by missense and nonsense POU1F1s. (**A**) In the TSHβ promoter, POU1F1 on the POU1F1-US sequence cooperates with GATA2, while that on the POU1F1-like sequence protects the function of GATA2 from inhibition by the suppressor region binding protein (SRBP) (de-repression). (**B**) The protein stability of six out of 15 mutants was reduced (Figure 1B). Therapy with recombinant GH (rGH) may decrease TSH production via type 2 deiodinase (D2), making mild TSHD overt. In the pathophysiology of TSHD caused by mutant POU1F1s, the impairment in de-repression against the SR plays a more critical role than the cooperation of POU1F1 on POU1F1-US with GATA2. The interaction of SRBP with the SR may also be involved in the distinct behaviors of K216E and R271W in the regulation of the TSHβ gene.

The results are summarized in Table 1, and the functions of the wild-type and mutant POU1F1 in TSHβ gene expression are reviewed in Figure 8. To the best of our knowledge, this is the first study to evaluate the transactivation functions of various mutant POU1F1s in the context of the TSHβ gene using a common experimental platform (Figure 2). We also compared them with those of the PRL promoter and found a correlation between them (Figure 3). Our study suggests the following possibilities: firstly, the protein stability of mutant POU1F1s plays an important role in the pathophysiology of POU1F1-related CPHD; secondly, rGH therapy may have implications for mild or delayed TSHD cases; thirdly, the interaction of SRBP with the SR is thought to be critical in the defects caused by mutant POU1F1s in TSHβ transcription; finally, the behaviors of K216E and R271W are distinct regarding the regulation of the TSHβ and PRL genes.

In our classification (Figure 3), only P14L [68] belongs to group I. Heterozygotes harboring P14L mutations have been reported to exhibit TSHD, GHD, and PRLD. Unexpectedly, however, the mother and grandmother were endocrinologically normal, although they were also heterozygous for this mutation [68]. Moreover, Kishimoto et al. [51] reported that the transactivation of the GH and PRL genes by this mutant was equivalent to that of the wild type, at least in COS7 cells. In the present study, the transactivation of the TSHβ promoter by this mutant in CV-1 cells was also comparable to that by the wild-type POU1F1 (Figure 2 and Figure 3).

Group II mutants comprised P24L, P76L, F135C, A158P, K216E, E230K, and R271W. These mutants possess partial transcriptional activity in the TSHβ and PRL promoters. Kishimoto et al. [51] suggested that the interaction between P24L and CBP is impaired, resulting in the decreased transactivation of the PRL promoter. However, the decreased protein levels of this mutant (Figure 1C) may also affect its transactivation function. In some patients with group II mutations, TSHD was detected following the diagnosis of GHD and rGH therapy [53,69]. Although the effect of GH should be experimentally determined in each mutation in the future, care should be taken in patients with group II mutations, where incomplete TSHD may be overlooked before rGH treatment. In addition, these patients should be carefully followed up with after rGH treatment [35,36,37].

In the group II mutants, the transactivation of the rat PRL promoter by K216E was low, while that of the TSHβ promoter was as potent as in the wild type (Figure 3). Al-Samerria et al. recently generated K216E knock-in mice [70]. As expected, the expression levels of GH and PRL were significantly lower in homozygous mice than in wild-type mice. Wild-type POU1F1 activates the transcription of its own gene (autoregulation) in cooperation with the retinoic acid receptor (RAR). Based on this, Cohen et al. [54] previously suggested that, since K216E has a defect in synergism with the RAR, its own expression may be decreased, resulting in a reduction in the expression of the downstream genes encoding TSHβ, GH, and PRL. In addition, the nuclear localization signal (NLS) of K216E may be impaired because codon 216 is located in its amino acid sequence (Figure 1A and Figure 7A) [71]. However, these findings do not explain the high transactivation of the TSHβ promoter by this mutant. The current and previous studies indicate that K216E is able to form monomers and dimers on the POU1F1-RE in the PRL gene, as in the case of the wild type (Figure 6A, lane 9) [54,58], and that lysin at 216 may not be involved in direct interaction with Ets [20]. Therefore, one of the reasons for the attenuated transactivation of the PRL promoter may be a defect in the interaction of K216E with the CREB-binding protein [54] on its POU1F1-REs.

Further investigation is required to understand the mechanism by which the K216E mutant is able to transactivate the TSHβ gene without the binding of the POU1F1-REs in this promoter (Figure 6B–D), in addition to the reduced interaction with GATA2 (Figure 7). Because K216E mutants have defects in binding to hTSHβ-PG or the SR (Figure 6B,C, lanes 11), complex formation of the K216E mutant with cofactors (or chromatin-modifying enzymes) on these elements is unlikely. The analysis of TSHβ-M3-CAT (Figure 4) suggested that the defect in DNA binding by POU1F1 with the SR plays a more important role than that with POU1F1-US in the pathogenesis of TSHD in POU1F1-related CPHD. We measured the transactivation of TSHβ-M3-CAT (Figure 4A) by K216E and found that the ratio was 1.39 ± 0.11, which is comparable to that in TSHβ-CAT (1.24 ± 0.07, calculated from the data in Figure 2). Thus, the K216E mutant is able to transactivate the TSHβ promoter, even in the presence of the SR. Because the signal for SRBP in the presence of K216E is almost abolished (Figure 6C, lane 9), we speculate that K216E may sequester SRBP from binding with the SR (Figure 8B). If this is the case, K216E may be a gain-of-function mutation. Interestingly, the K216E mutation may generate a de novo small ubiquitin-like modifier (SUMO) site, as predicted by three programs, namely GPS-SUMO [72], SUMOplot (https://www.abcepta.com/sumoplot), and DeepSUMO (http://deepsumo.renlab.org). However, further investigation, including the identification of SRBP, is required in the future. Surprisingly, the TSHβ mRNA and serum TSH concentrations in K216E knock-in homozygous mice were approximately two-fold higher than in wild-type mice in spite of normal T3 and T4 levels [70]. As suggested by the authors, this observation may be specific to mice. For example, an alternative splicing variant of POU1F1, Pit-1T, was detected in the mouse pituitary gland. Although Pit-1T has a stronger transactivation function than wild-type POU1F1 [73], its expression was absent in the pituitary in rhesus monkeys [74] or rat somatotroph cell lines (GH3 or GH4) [75].

Aside from the mechanism of the transactivation of the TSHβ promoter by the K216E mutant, the protein–protein interactions of POU1F1 with GATA2 have been postulated to play certain roles in the transactivation of the TSHβ promoter [44,45]. Unexpectedly, however, our results for the K216E mutant (Figure 7C) suggest the possibility that this interaction is neutral, at least in the transcription of the TSHβ promoter (Figure 3), although we cannot completely exclude the possibility of an unknown indirect mechanism for this mutation. On the other hand, the physical association of POU1F1 with GATA2 has been thought to interfere with the expression of the SF-1 transcription factor [45], a critical determinant of gonadotroph development in the pituitary gland. Interestingly, Al-Samerria et al. reported a lack of sexual activity in male and female mice harboring a K216E mutation [70].

The transactivation of the rat PRL promoter by R271W [41] was the highest with group II mutants, while that of the TSHβ promoter was as low as in the group III mutants (Figure 3). This mutant has been reported to fail to associate with the nuclear matrix [76] and to induce apoptosis in GH3 cells [77]. Nonetheless, these findings do not explain the TSHβ gene-specific reduction in transcription compared with the PRL gene (Figure 3) and the high frequency of the TSHD-dominant phenotype in patients with this mutation. Moreover, it is unknown why the defect in R271W in the transactivation of the PRL promoter is modest, at least in HeLa (this study), COS7, JEG3, and CHO cells [78]. The results in Figure 6A and previous reports [54] indicate that the monomer—but not dimer—formation of R271W is maintained via rPRL-1P or other POU1F1-REs in the PRL gene (Figure 2, top left). Therefore, the POU1F1 monomer may be able to partially transactivate this promoter. The R271W mutant has reduced binding with either hTSHβ-PG or the SR (Figure 6B,C, lanes 11). In contrast, among the six mutants examined in Figure 6C, the signal for SRBP (Figure 6C, lane 12) is the highest in the presence of R271W (lane 11). Therefore, the defective DNA binding by the R271W mutant and the persistent occupancy of the SR by SRBP may be the major reasons for the TSHβ gene-specific reduction in transactivation (Figure 8B). Although SRBP binding was also detected in the presence of wild-type POU1F1 (Figure 6C, lane 3), the binding of wild-type POU1F1 with hTSHβ-PG and the SR (Figure 6B,C, lane 3) was much stronger than that of the R271W mutant (Figure 6B,C, lane 11). Thus, the relatively potent binding of wild-type POU1F1 with these elements may overcome the inhibitory effects of SRBP on the SR.

The transcriptional activity of group III mutants was equally impaired in both the TSHβ and PRL promoters. In all group III mutations examined, the inheritance of POU1F1-related CPHD was reported to be homozygous or compound heterozygous. The protein expression levels of F233S, E250X, and K145X were reduced (Figure 1B). In agreement, the inheritance of the patient with E250X was reported to be homozygous [79]; however, the heterozygotes for this mutation are reported to result in relatively short stature. Similarly, short stature and mild CPHD have also been reported in heterozygotes for Q4X [80] and K145X [81] mutations, respectively. These findings suggest a dosage effect [81] or haploinsufficiency in the pathophysiology of POU1F1-related CPHD.

The following questions remain unanswered. First, due to a lack of appropriate cell lines, which are not only transfectable but also able to mimic the thyrotroph, we employed here a reconstitution system with mouse kidney-derived CV-1 cells, where POU1F1 and GATA2 were co-transfected [46,82]. Low T3 concentrations in patients with TSHD increase TRH secretion via the H-P-T axis (Figure 8), which subsequently increases the expression of the TSHβ and PRL genes [83]. Our reconstitution system is a useful tool to examine the influence of the inhibition of the TSHβ gene by T3 [46,82] and the activation by TRH [40]. However, in the pituitary gland, intracellular negative regulation by T3 has been reported in the D2 [84], GATA2 [85], POU1F1 [86], and TRH receptor genes [87,88]. Although there are some cell lines derived from lactotrophs (e.g., GH3 cells), we did not utilize such cells because the endogenous POU1F1 expression may complicate the experimental conditions. However, there are complex intercellular communications among pituitary hormone-producing cells [45]. Thus, in the future, in vivo analyses of mutant POU1F1s are necessary, although there may be a difference between human and mouse cases, as in the case with K216E knock-in mice [70]. Second, we utilized a relatively short TSHβ promoter construct (−128/+37). Although we previously analyzed a longer TSHβ promoter construct (−1193 to +37) in CV-1 cells, we found that not only the transactivation by POU1F1 and GATA2 but also the T3 and TRH responsiveness were similar to those of the short construct [40,82]. Using next-generation sequencing (NGS), the mouse TSHβ gene in TαT1 cells was recently analyzed [89]. The authors reported a new POU1F1-RE, element 4, between 7.7 and 6.6 kb upstream of the transcription start site, and they examined the function of this element fused with the proximal promoter (438 bp) using CV-1 cells. However, in the presence of GATA2, no additive or synergistic effect was observed upon the overexpression of POU1F1, and its transcriptional activity was comparable to that of the proximal promoter only. In this study, we used a rat-derived 0.6 kb PRL promoter construct [51] (Figure 2, top left). This construct has been well characterized [51], and the transcriptional activity of the chromatinized DNA of this region is comparable to that of a 1.9 kb promoter region [90]. However, further investigation is necessary because the human PRL genome is predicted to harbor more than 18 POU1F1-REs within a 5.8 kb DNA region [13]. Third, the fact that patients with P14L, P24L, P76L, K216E, and R271W mutants were reported to be heterozygotes (shown in italics in Figure 1, Figure 2, Figure 3, Figure 4 and Figure 6) [91] suggests the dominant-negative effect (DNE) of these mutants. It was speculated that the positions of DNE mutations may be located in a codon outside the POU_S_ or POU_H_ domain (Figure 1A) [80]. However, further studies are required, particularly on the mechanism(s) of DNE by R271W and K216E mutants, which fail to bind the POU1F1-US (Figure 6B) or POU1F1-like sequence/SR but influence SRBP binding (Figure 6C).

## 4. Materials and Methods

### 4.1. Plasmid Construction

TSHβ (−128/+37)-CAT was constructed by fusing the human TSHβ promoter (nt. −128/+37) to the CAT reporter gene, as described previously [46]. TSHβ-M3-CAT has been reported elsewhere [6]. The 0.6 kb PRL-Luc, which contained the 0.6 kb rat PRL 5′-flanking region and firefly luciferase coding sequence, was gifted by Dr. Yasuhiko Okimura (Kobe Women’s University, Kobe, Hyogo, Japan). The expression plasmids for human POU1F1 (pCB6^+^-hPIT1) and mouse GATA2 (pcDNA3-mGATA2) have been described previously [6]. Mutant human POU1F1 expression plasmids carrying P14L, P24L, P76L, F135C, K145X, A158P, R172Q, S179R, W193R, K216E, E230K, F233S, P239S, E250X, or R271W were generated by site-directed mutagenesis with specific primers using the PrimeSTAR Mutagenesis Basal Kit (Takara Bio, Shiga, Japan). All mutated sequences were confirmed by Sanger sequencing.

### 4.2. Cell Culture and Transient Transfection

CV-1 and HeLa cells were grown in a monolayer culture at 37 °C in carbon dioxide (CO2)/air (1:19) in Dulbecco’s modified Eagle’s medium containing 10% (*v*/*v*) fetal calf serum (FCS), penicillin (100 units/mL), streptomycin (100 μg/mL), and amphotericin B (0.25 µg/mL). All cells were trypsinized and plated in 12-well plates for 24 h before transient transfection. CV-1 and HeLa cells at a density of 0.5 × 10^5^ and 1.0 × 10^5^ cells per well were transfected using Lipofectamine 3000 reagent (Invitrogen, Waltham, MA, USA), according to the manufacturer’s instructions. After the cells had been exposed to Lipofectamine 3000 reagent for 24 h, the medium was replaced with fresh medium containing 10% (*v*/*v*) FCS. After incubation for 24 h, the cells were harvested. CAT activity was measured as previously described [92]. Luciferase activity was measured using a PicaGene Luminescence Kit (TOYO-B-NET, Tokyo, Japan). CAT and luciferase activity was normalized by β-galactosidase activity. For each reporter assay, cells were transfected with pCMV-CAT or pRSV-Luc, the magnitudes of which were adjusted to 100%.

### 4.3. Western Blotting Analysis

CV-1 cells were transfected with pCB6^+^-hPIT1 wild type, P14L, P24L, P76L, F135C, K145X, A158P, R172Q, S179R, W193R, K216E, E230K, F233S, P239S, E250X, or R271W (7.5 μg per 6 cm dish). After incubation for 24 h, the cells were harvested and whole-cell extracts were fractionated by sodium dodecyl sulfate–polyacrylamide gel electrophoresis (SDS-PAGE) and transferred to polyvinylidene difluoride membranes (Millipore, Burlington, MA, USA). The membranes were blocked with 5% skim milk in PBST buffer (PBS with 0.05% Tween-20) for 1 h at room temperature. After washing in PBST buffer, the membranes were incubated with anti-PIT1 N-terminus antibody (sc-393943, Santa Cruz Biotechnology, Dallas, TX, USA; diluted 1:500) or anti-PIT1 C-terminus antibody (GTX77853, GeneTex, Irvine, CA, USA; diluted 1:500) overnight at 4 °C. After washing in PBST buffer, the membranes were incubated with anti-mouse (NA931, Cytiva, Tokyo, Japan; diluted 1:1000) or anti-rabbit (NA934, Cytiva, Tokyo, Japan; diluted 1:1000) secondary antibody for 1 h at room temperature. Bands were detected using the ECL Prime Western Blotting Detection Reagent (RPN2232, Cytiva, Tokyo, Japan) and the FUSION FX7 system (Vilber Lourmat, Marne-la-Vallée, France).

### 4.4. Gel Shift Assay

Double-stranded oligo DNA for the hTSHβ-PG-probe (sense: 5′-AGTATGAATTTTCAATAGATGCTTTTCAGATAAGAAA-3′ and antisense: 5′-TTTCTTATCTGAAAAGCATCTATTGAAAATTCATACT-3′), hTSHβ-SR-probe (sense: 5′-AGTGAATCAAATGCAATTGTATAAACAAGAAGATC-3′ and antisense: 5′-GATCTTCTTGTTTATACAATTGCATTTGATTCACT-3′), hTSHβ-PGSR-probe (sense: 5′-AGTATGAATTTTCAATAGATGCTTTTCAGATAAGAAAGCAGTGAATCAAATGCAATTGTATAAACAAGAAGATC-3′ and antisense: 5′-GATCTTCTTGTTTATACAATTGCATTTGATTCACTGCTTTCTTATCTGAAAAGCATCTATTGAAAATTCATACT-3′), and rPRL-1P-probe (sense: 5′-TGCCTGATTATATATATATTCATGAAGGTGTCG-3′ and antisense: 5′-CGACACCTTCATGAATATATATATAATCAGGCA-3′) was labeled with ^32^P-ATP using T4 polynucleotide kinase (Takara Bio, Shiga, Japan). CV-1 cells were transfected with pCB6^+^-hPIT1 wild type, P76L, A158P, R172Q, K216E, E230K, R271W, or pcDNA3-mGATA2 (15 μg per 10 cm dish). After incubation for 24 h, the medium was replaced with a fresh medium containing 10% (*v*/*v*) FCS. After incubation for an additional 24 h, the cells were harvested and nuclear extracts were prepared as previously described [46]. ^32^P-labeled probes and nuclear extracts were incubated for 20 min at room temperature in 20 μL of binding buffer containing 20 mM HEPES-NaOH (pH 7.9), 50 mM KCl, 0.1 mM ethylenediaminetetraacetic acid, 10% (*v*/*v*) glycerol, 0.5 mM dithiothreitol, and 2 μg of poly (dI-dC). DNA–protein complexes were resolved by electrophoresis on a 5% (*w*/*v*) polyacrylamide gel at 150 V for 130 min at 4 °C. For the super-shift assay, anti-PIT1 C-terminus antibody (GTX77853, GeneTex, Irvine, CA, USA) was mixed with the PIT1 protein and incubated for 3.5 h at 4 °C before incubation with ^32^P-labeled probes. The gel was dried and the labeled bands were visualized using an Amersham Typhoon scanner IP system (Cytiva, Tokyo, Japan).

### 4.5. GST Pulldown Assay

*Escherichia coli* (DH5α) that had been transformed with pGEX-4T-2-hGATA2 (full length) was induced with 2 mM isopropyl-β-D-thiogalactopyranoside for 4 h. The *E. coli* pellet was sonicated, and the fusion proteins were purified by mixing with glutathione Sepharose 4 B (Cytiva, Tokyo, Japan, Figure 7B), R172Q, K216E, and E230K. POU1F1 proteins were translated in vitro using rabbit reticulocyte lysates (Promega Corporation, Madison, WI, USA) in the presence of ^35^S-methionine. Radiolabeled POU1F1s were incubated with GST-GATA2 fusion proteins (Figure 7A bottom) in binding buffer [150 mM NaCl, 20 mM Tris-HCl (pH 7.5), 1% Triton X-100, 1 mM dithiothreitol, 0.5 mM phenylmethylsulfonyl fluoride, 0.1 μg/mL leupeptin, 1 μg/mL aprotinin] for 3 h at 4 °C and washed three times with the binding buffer. Bound proteins were analyzed using 12% SDS-PAGE and visualized using an Amersham Typhoon scanner IP system (Cytiva, Tokyo, Japan).

### 4.6. Statistical Analysis

Each reporter assay was performed in duplicate more than three times, and each result was expressed as the mean ± standard error (SE). Statistical significance was examined using analysis of variance and Fisher’s protected least significant difference test using the StatView software (version 4.0; Abacus Concepts, Berkeley, CA, USA). Statistical significance was set at a *p*-value of less than 0.05.

## 5. Conclusions

NGS has facilitated the detection of genetic mutations [93]. However, efforts to compare the functions of mutant POU1F1 proteins as a spectrum in the context of different target genes are still necessary because these mutant proteins are predicted to have functional diversity, owing to their flexible protein structures and the differences in partner transcription factors specific to the target genes. In particular, TSHD is one of the most critical phenotypes of POU1F1-related CPHD, and it should be diagnosed as early as possible to prevent mental retardation [94]. Because the transactivation levels of mutant POU1F1s, including K216E and R271W (Figure 3), seem to reflect their clinical profiles, similar approaches may provide useful information for their diagnosis and prognosis.

## Figures and Tables

**Table 1:** A summary of mutant POU1F1s for which DNA binding (Figure 6) and/or protein–protein interaction with GATA2 (Figure 7) were examined. The mutants whose inheritance is reported to be heterozygotic are indicated in italic. The protein level or the potency of interactions with DNA, GATA2, or the CREB-binding protein (CBP) equivalent to wild-type POU1F1 is indicated as +++. M, monomer-dominant; ND, not determined. The interaction between mutant POU1F1 and CBP was inferred from previous reports from Cohen et al. [54].

POU1F1 Mutation	Group	Protein Level	DNA Binding					Protein–Protein Interaction	
			rPRL-1P	hTSHβ					
				PG	SR		PGSR	GATA2	CBP
			POU1F1			SRBP	POU1F1-GATA2		
Wild	I	+++	+++			++	+++	+++	
*P76L*	II	+++	+	+/−		+/−	ND	ND	
A158P	II	++	++	+/-		+/−	ND	ND	+
R172Q	III	++	−			+/−	−	+/−	ND
*K216E*	II	++	+++	−		−	−	+/−	
E230K	II	+++	M+	+/−		+/−	−	+++	ND
*R271W*	II	+++	M++	−		+	ND	ND	+++
Figure	3	1B	6A	6B	6C		6D	7C,D	([54])

## Data Availability

The original contributions presented in this study are included in the article/Supplementary Materials. Further inquiries can be directed to the corresponding author.

## Supplementary Materials

The following supporting information can be downloaded at: https://www.mdpi.com/article/10.3390/ijms27010119/s1.

## Abbreviations

The following abbreviations are used in this manuscript:

BSA	bovine serum albumin
CAT	chloramphenicol acetyltransferase
CBP	CREB-binding protein
CPHD	combined pituitary hormone deficiency
CREB	cAMP response element-binding protein
D2	type 2 deiodinase
DNE	dominant-negative effect
Ets	E26 transformation-specific transcription factor
FCS	fetal calf serum
GATA2	GATA-binding protein 2
GATA-RE	GATA-responsive element
GH	growth hormone
GHD	defects in the production of GH
GST	glutathione S-transferase
H-P-T	hypothalamus–pituitary–thyroid
Luc	luciferase
NCoR	nuclear receptor co-repressor
NGS	next-generation sequencing
P/CAF	p300/CBP-associated factor
POU1F1-RE	POU1F1-responsive element
POU1F1-US	POU1F1-RE upstream
POU1F1-like	POU1F1-RE-like sequence
POUH	POU homeodomain
POUS	POU-specific domain
PRL	prolactin
PRLD	defects in the production of PRL
RAR	retinoic acid receptor
rGH	recombinant GH
SDS-PAGE	sodium dodecyl sulfate–polyacrylamide gel electrophoresis
SR	suppressor region
SRBP	suppressor region binding protein
SUMO	small ubiquitin-like modifier
T3	triiodothyronine
T4	thyroxine
TR	T3 receptor
TRH	thyrotropin-releasing hormone
TSH	thyroid-stimulating hormone
TSHD	defects in the production of TSH
TSS	transcription start site
Znf	zinc finger
