# Supplementary material for: Functional Properties of POU1F1 Mutants in the Transcriptional Regulation of the Thyrotropin β Gene Compared with the Prolactin Gene
Source: Int J Mol Sci. 2025 Dec 22;27(1):119. doi: 10.3390/ijms27010119 (PMC12785802; doi:10.3390/ijms27010119)
Supplement: Supplementary file 1 [file ijms-27-00119-s001.zip › ijms-3939821-supplementary.pdf]

primers for mutagenesis of human POU1F1

PIT1-P14L-sense:	5′-TTTATACTTCTGAATTCTGACGCCTCTGC-3′
PIT1-P14L-antisense:	5′-ATTCAGAAGTATAAAGGTATCAGCCGAAG-3′
PIT1-P24L-sense:	5′-ACTCTGCTTCTGATAATGCATCACAGTGC-3′
PIT1-P24L-antisense:	5′-TATCAGAAGCAGAGTTGCAGAGGCGTCAG-3′
PIT1-P76L-sense:	5′-TTAACCCTTTGTCTTTATAAATTTCC-3′
PIT1-P76L-antisense:	5′-AAGACAAAGGGTTAAACTACCTGCCATCACTC-3′
PIT1-F135C-sense:	5′-GAAAAGTGTGCCAATGAATTTAAAGTG-3′
PIT1-F135C-antisense:	5′-ATTGGCACACTTTTCAAGTTCTCTGATTTC-3′
PIT1-K145X-sense:	5′-ACGAATTTAATTAGGATACACCCAGAC-3′
PIT1-K145X-antisense:	5′-CCTAATTAAATTCGTCTCACTTTAAATTC-3′
PIT1-A158P-sense:	5′-GGCCCTGCCAGCTGTGCATGGCTCTGAATTCAGTC-3′
PIT1-A158P-antisense:	5′-ACAGCTGGCAGGGCCTCCCCAACATTTGTC-3′
PIT1-R172Q-sense:	5′-ATCTGCCAATTTGAAAATCTGCAGCTCAG-3′
PIT1-R172Q-antisense:	5′-TTCAAATTGGCAGATTGTTGTTTGAC-3′
PIT1-S179R-sense:	5′-AGCTCAGGTTTAAAAATGCATGCAAAC-3′
PIT1-S179R-antisense:	5′-TTTTAAACCTGAGCTGCAGATTTTCAAATCG-3′
PIT1-W193R-sense:	5′-ATCCAAACGGCTGGAGGAAGCTGAGCAAG-3′
PIT1-W193R-antisense:	5′-TCCAGCCGTTTGGATAATATTGCTTTC-3′
PIT1-K216E-sense:	5′-GAAAAGAGAACGAAGAACAACTATAAG-3′
PIT1-K216E-antisense:	5′-CTTCGTTCTCTTTTCCTTTCATTTGCTC-3′
PIT1-E230K-sense:	5′-TGCTCTGAAGAGACACTTTGGAGAAC-3′
PIT1-E230K-antisense:	5′-TGTCTCTTCAGAGCATCTTTAGCAGC-3′
PIT1-F233S-sense:	5′-AGACACTCTGGAGAACAGAATAAACC-3′
PIT1-F233S-antisense:	5′-TTCTCCAGAGTGTCTCTCCAGAGCATC-3′
PIT1-P239S-sense:	5′-GAATAAATCTTCTTCTCAAGAGATCATGAGG-3′
PIT1-P239S-antisense:	5′-GAAGAAGATTTATTCTGTTCTCCAAAG-3′
PIT1-E250X-sense:	5′-GGCTGAATAACTGAATCTGGAGAAAG-3′
PIT1-E250X-antisense:	5′-TTCAGTTATTCAGCCATCCTCATGATCTC-3′
PIT1-R271W-sense:	5′-AGAAAAATGGGTGAAAACAAGTCTGAATC-3′
PIT1-R271W-antisense:	5′-TTCACCCATTTTTCTCTCTGCCTCCGGTTG-3′

Supplementary Table

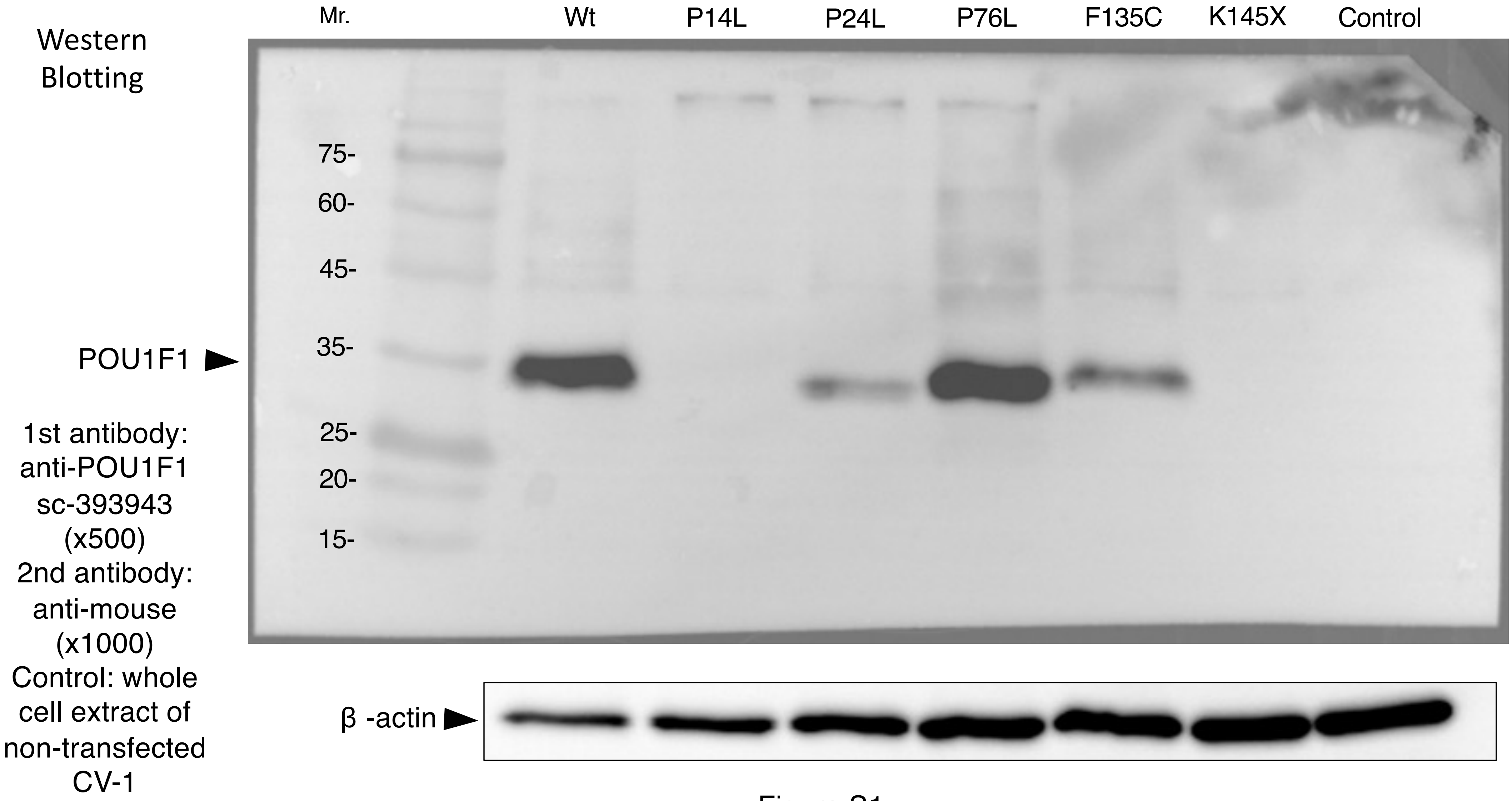
Western Blotting
Mr.
Wt
P14L
P24L
P76L
F135C
K145X
Control
75-
60-
45-
35-
25-
20-
15-
POU1F1
1st antibody: anti-POU1F1 sc-393943 (x500)
2nd antibody: anti-mouse (x1000)
Control: whole cell extract of non-transfected CV-1
β -actin

Figure S1

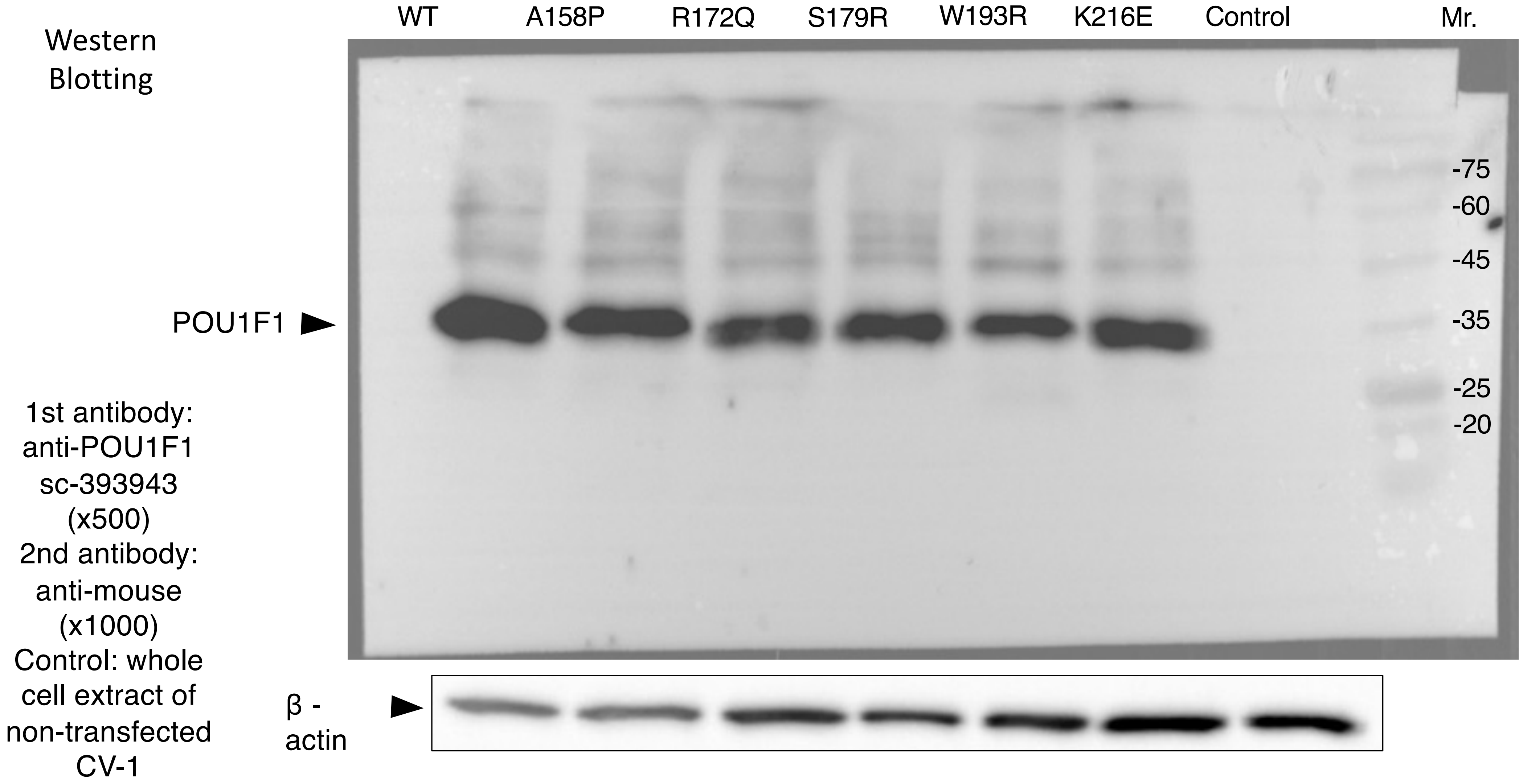
Western Blotting
WT
A158P
R172Q
S179R
W193R
K216E
Control
Mr.
-75
-60
-45
-35
-25
-20
POU1F1
1st antibody: anti-POU1F1 sc-393943 (x500)
2nd antibody: anti-mouse (x1000)
Control: whole cell extract of non-transfected CV-1
β - actin

Figure S2

Western Blotting

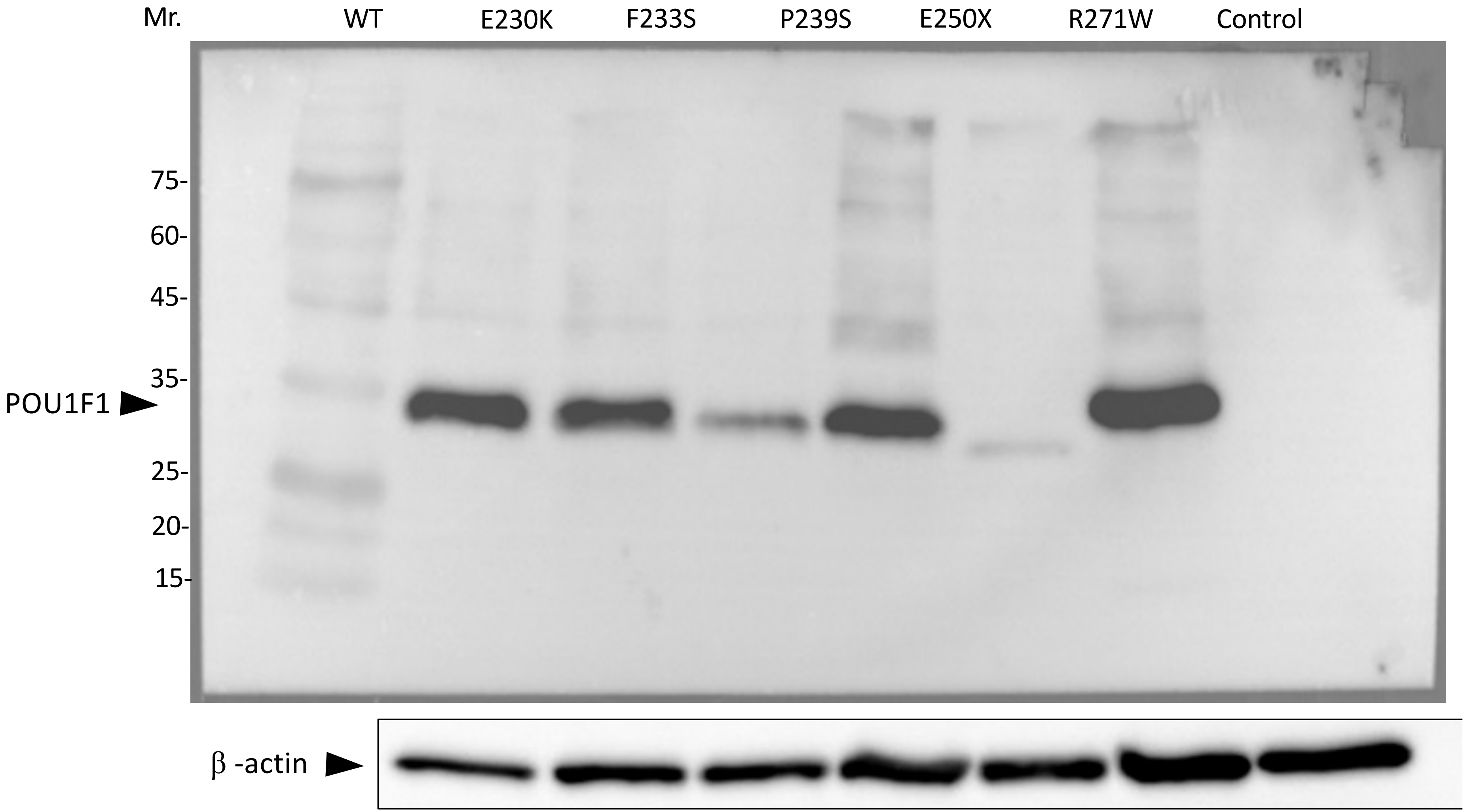

1st antibody: anti-POU1F1 sc-393943 (x500)
2nd antibody: anti-mouse (x1000)
Control: whole cell extract of non-transfected CV-1

Figure S3

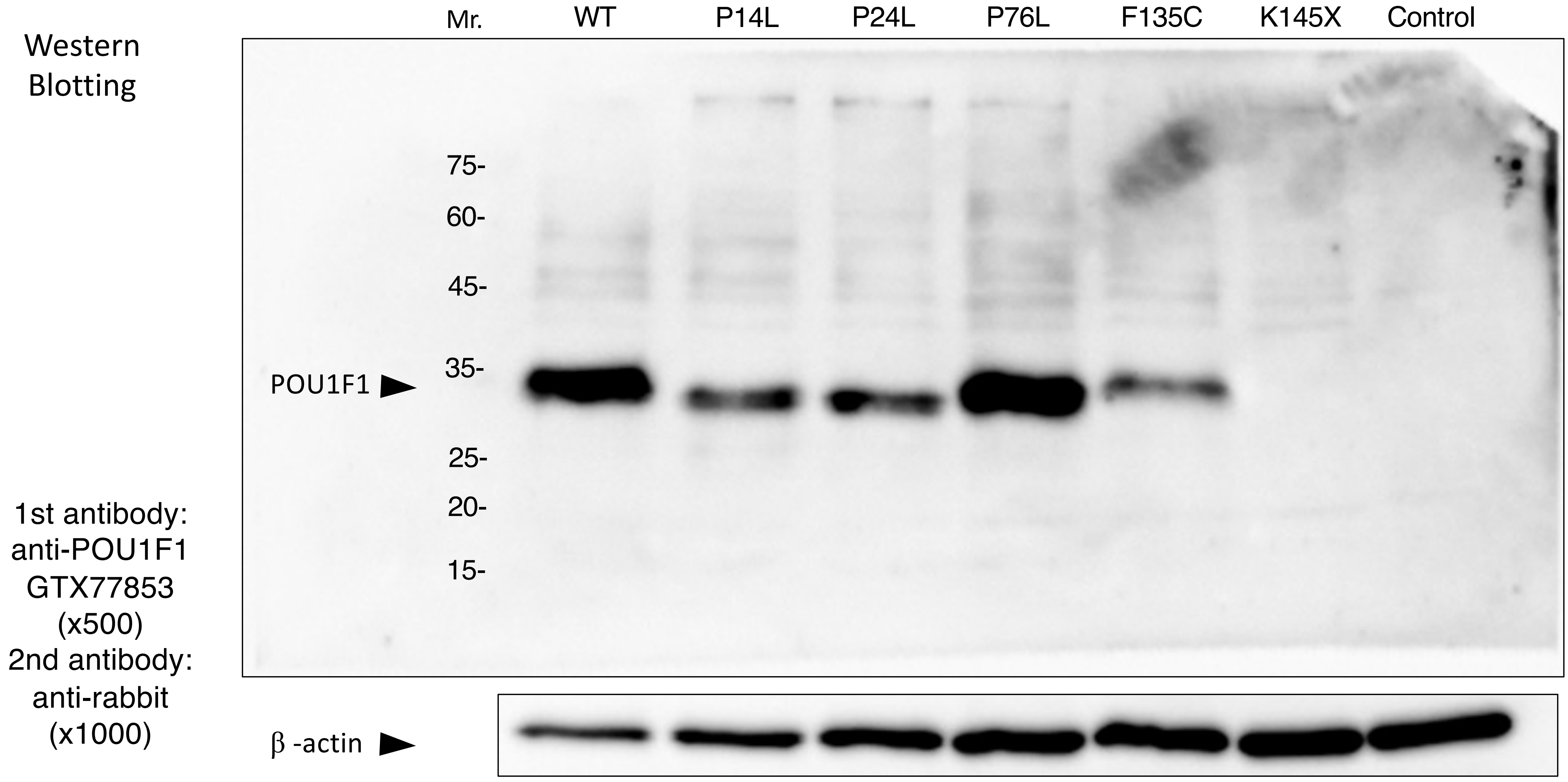

Western
Blotting
Mr.
WT
P14L
P24L
P76L
F135C
K145X
Control
75-
60-
45-
35-
25-
20-
15-
POU1F1
1st antibody:
anti-POU1F1
GTX77853
(x500)
2nd antibody:
anti-rabbit
(x1000)
β -actin

Figure S4

〈Gel shift assay〉
2023.12.29 - 12.22

Wt: wild-type
POU1F1
Ab: GTX77853

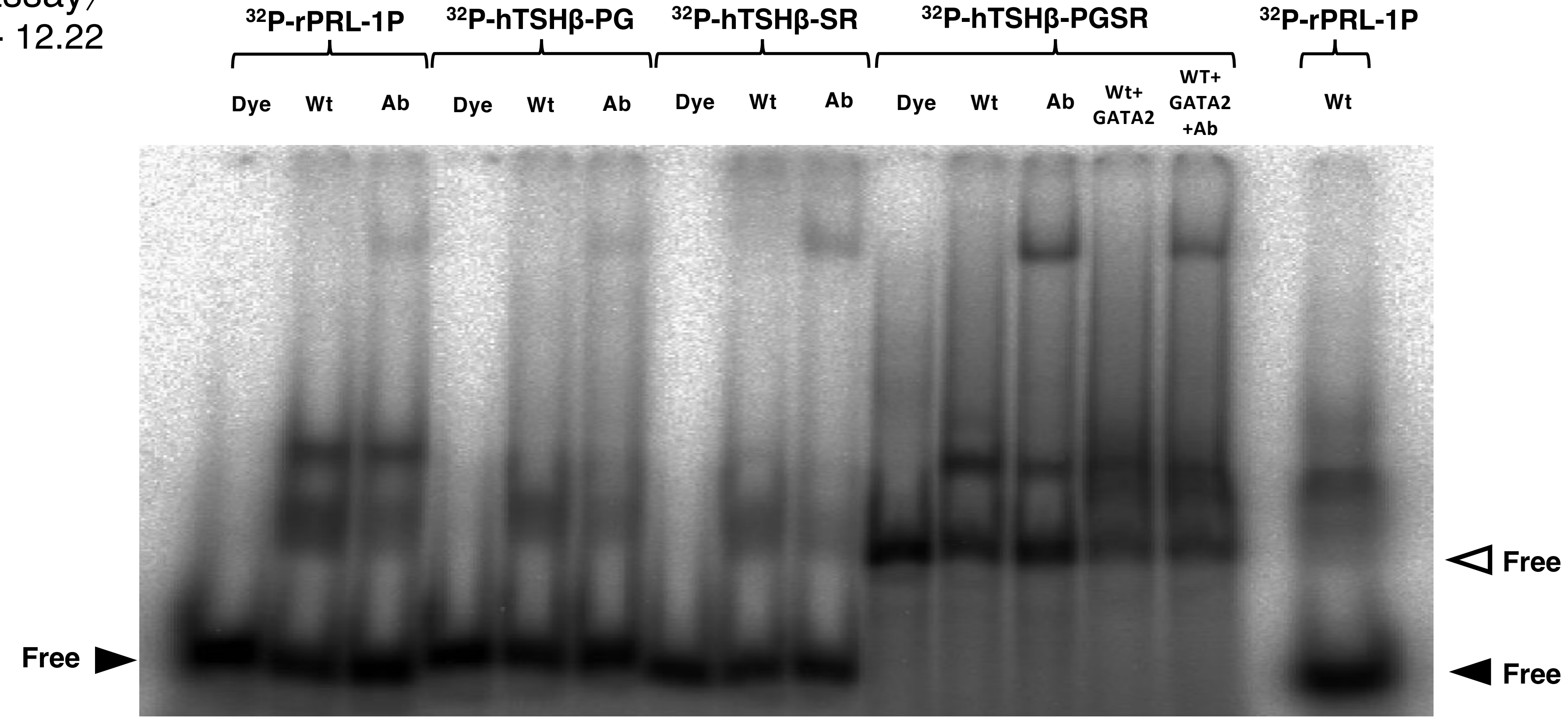

Figure S5

〈Gel shift assay〉
2023.10.13 - 10.11

Wt: wild-type POU1F1
NE: nuclear extract of non-transfected CV-1 cells
NS: non-specific cold
rPRL-1P: cold rPRL-1P

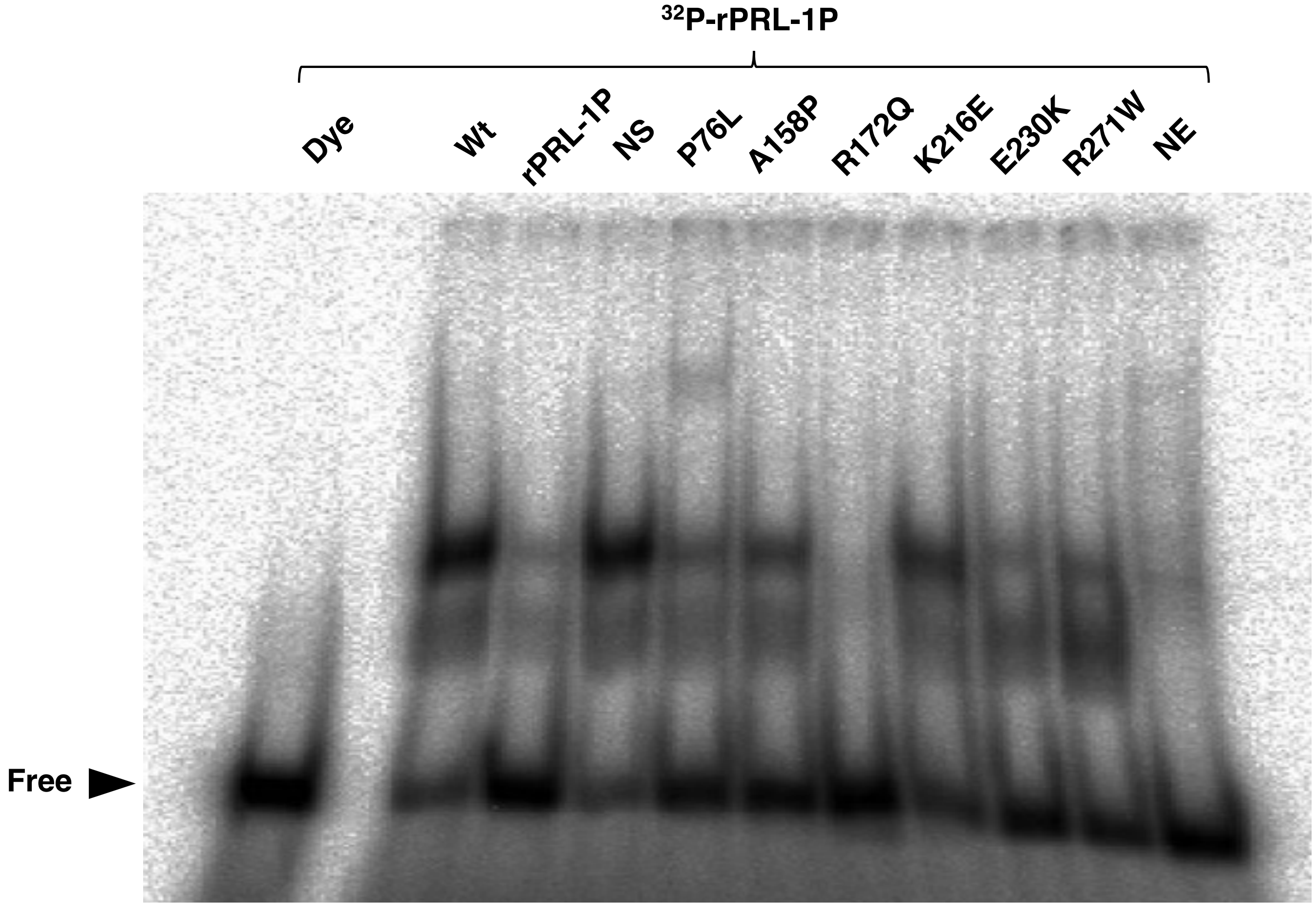

Figure S6

〈Gel shift assay〉
2023.10.25 - 10.26

Wt: wild-type POU1F1
NE: nuclear extract of non-transfected CV-1 cells
rWt: re-freezed wild-type POU1F1
NS: non-specific cold
PG: cold hTSHβ-PG
Ab: GTX77853

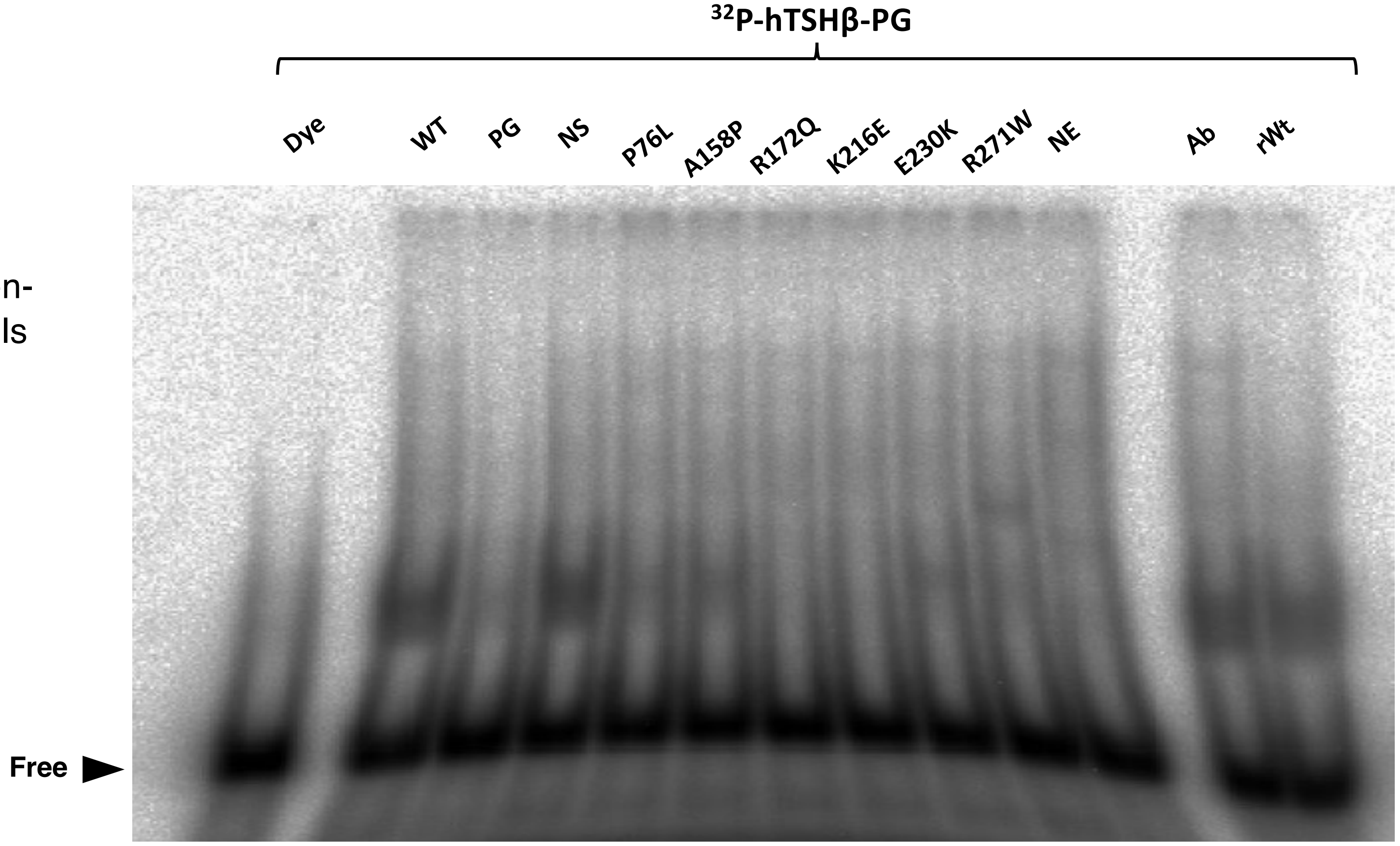

Figure S7

〈Gel shift assay〉
2023.10.25 - 10.26

Wt: wild-type POU1F1
NE: nuclear extract of non-transfected CV-1 cells
rWt: re-freezed wild-type POU1F1
NS: non-specific cold
SR: cold hTSHβ-SR
Ab: GTX77853

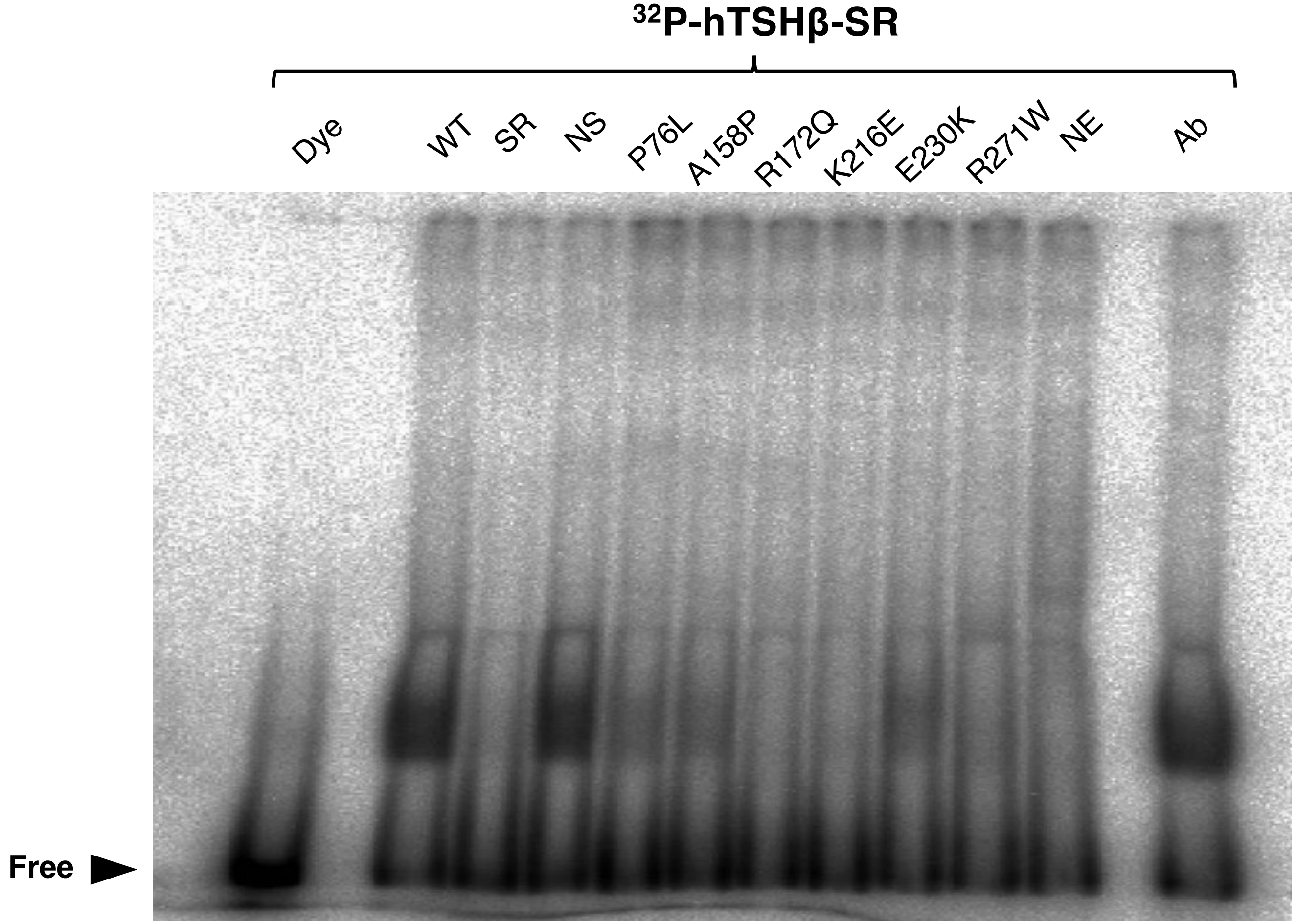

Figure S8

〈Gel shift assay〉
2023.12.13 - 12.15

NE: nuclear extract of non-transfected CV-1 cells
Wt: wild-type POU1F1
NS: non-specific cold
PGSR: cold hTSHβ-PGSR

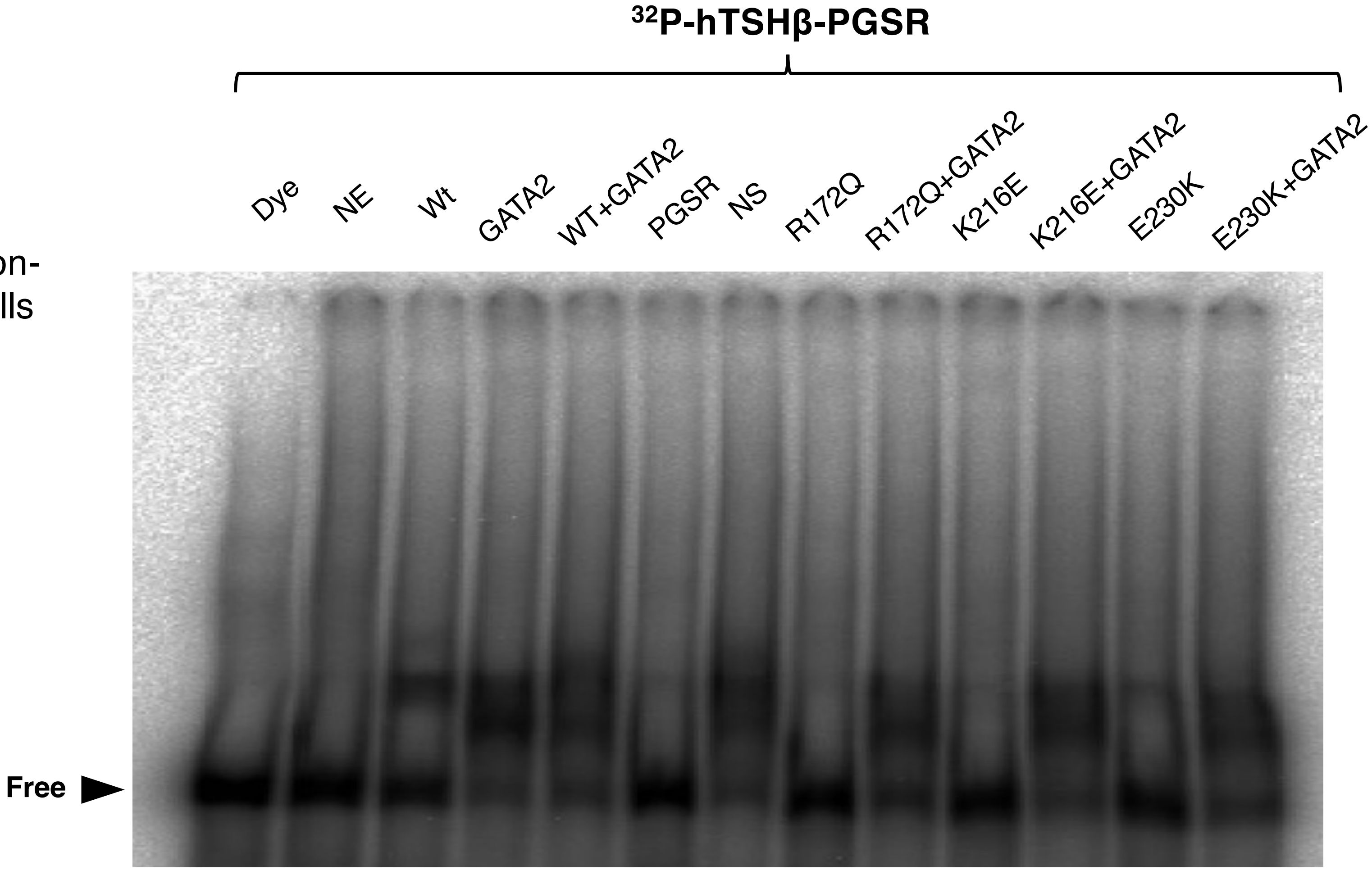

Figure S9

preparation of
GST-GATA2

SDA-PAGE
and Coomassie
blue stain

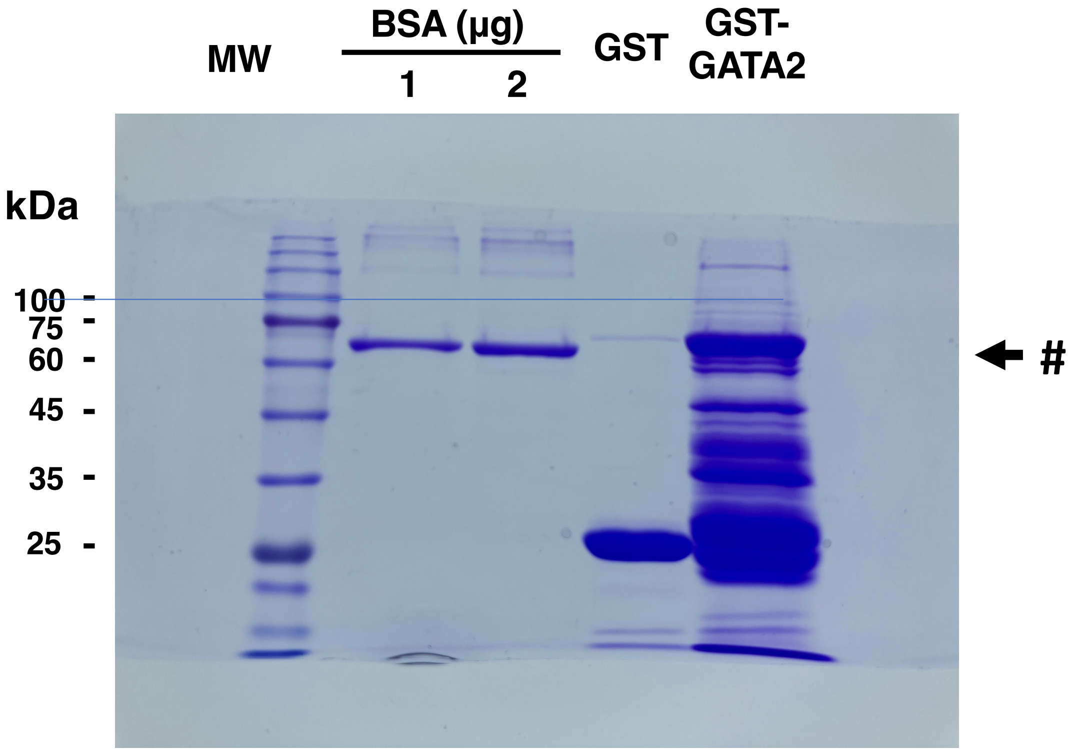

Figure S10

〈 GST pull-down assay 〉

2024.4.17-4.19

GST: GST alone

GST-GATA2:

GST fused with GATA2

Wt: wild-type POU1F1

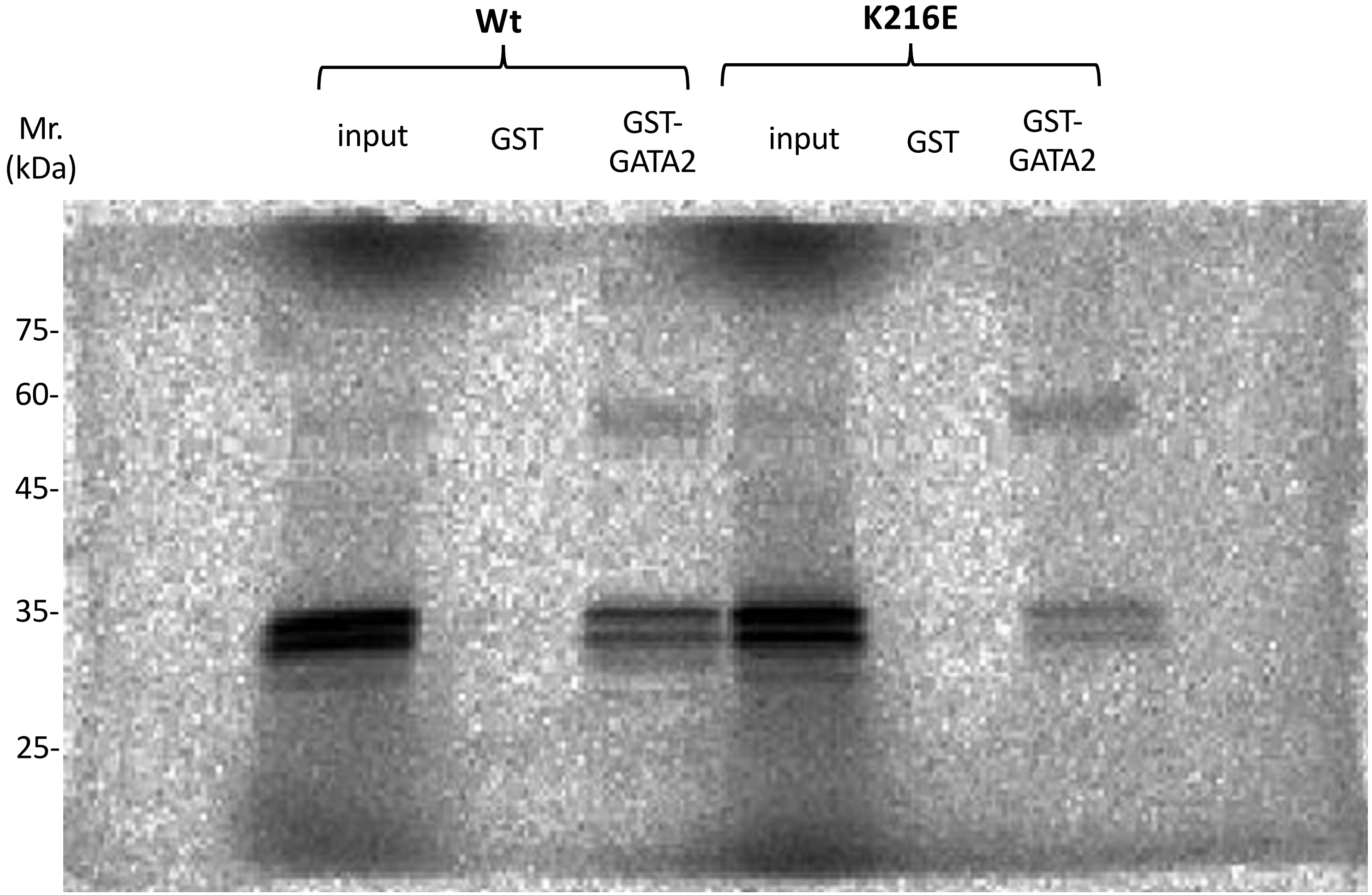

Figure S11

〈 GST pull-down assay 〉
2024.04.17-4.19

GST: GST alone
GST-GATA2:
GST fused with GATA2
Wt: wild-type POU1F1

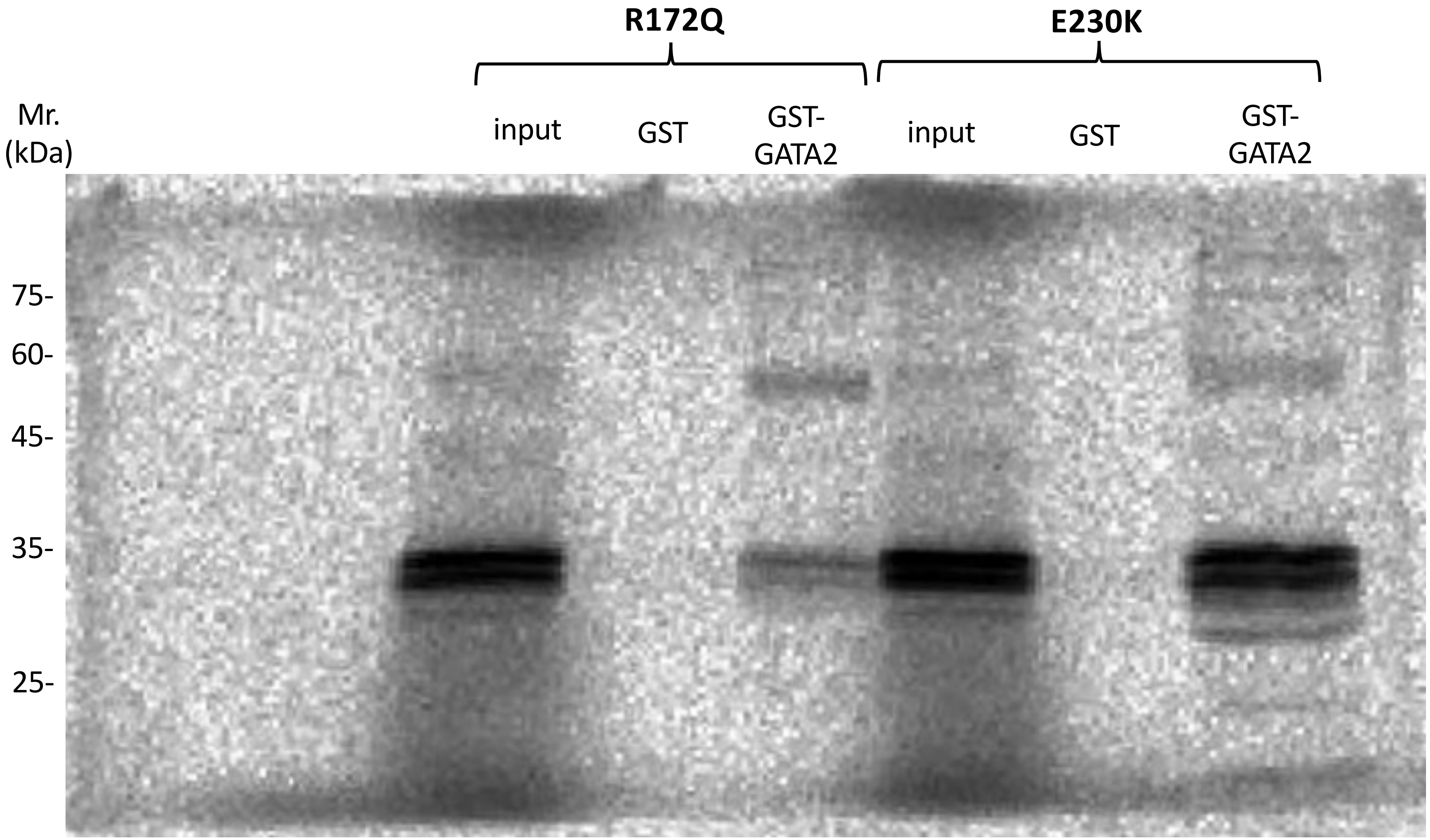

Figure S12
